# Trait‐Based Life History Strategies Shape Bacterial Niche Breadth

**DOI:** 10.1002/advs.202405947

**Published:** 2025-05-08

**Authors:** Ziheng Peng, Yiran Zhang, Xiaomeng Li, Hang Gao, Yu Liu, Yining An, Xun Qian, Gehong Wei, Shuo Jiao

**Affiliations:** ^1^ State Key Laboratory for Crop Stress Resistance and High‐Efficiency Production, Shaanxi Key Laboratory of Agricultural and Environmental Microbiology, College of Life Sciences Northwest A&F University Yangling Shaanxi 712100 P. R. China; ^2^ College of Natural Resources and Environment Northwest A&F University Yangling Shaanxi 712100 P. R. China

**Keywords:** life‐history strategies, metabolic module, niche breadth, resource acquisition, soil bacteria

## Abstract

The ecological niche represents a fundamental property of organisms, reflecting their diversity of utilized resources or environmental tolerances across space and time. Despite a wealth of studies revealing that not all bacteria being everywhere, the key traits that determine niche breadth have remained unclear. Here, bacterial niche breadth based on a large‐scale soil survey across a wide range of environmental gradients at a national‐scale is characterized, and evaluated their life‐history traits utilizing over 2000 bacterial genomic datasets from the Genome Taxonomy Database (GTDB). A positive relationship between gene functional diversity and niche breadth is found, and identified a key set of bacterial traits associated with niche breadth, which are assigned to five life‐history categories, encompassing growth, competition, stress tolerance, resource acquisition, and dispersal ability. The traits of these categories are captured by distinct clusters in the full dimensionality of trait space, suggesting that a broad‐niche taxon may indeed possess multiple facets of life history strategies essential for survival in diverse environments. Bacterial taxa with wider niche breadth maximized a diversity of traits associated with different life history strategies, whereas specialists tended to harbor a smaller number of traits associated with fewer life history strategies. Together, this study offers new insights into developing a trait‐based understanding of bacterial niche breadth from the perspective of life history theory.

## Graphical abstract

This study investigates bacterial niche breadth through a large‐scale soil survey and over 2000 bacterial genomes. It identifies key life‐history traits—growth, competition, stress tolerance, resource acquisition, and dispersal—that correlate with niche breadth. Generalists exhibit diverse life‐history strategies, while specialists tend to have fewer traits related to fewer strategies.

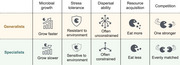

## 1 Introduction

The view of microbial biogeography patterns proposed by Baas‐Becking, hypothesizing that “everything is everywhere, but the environment selects”, is a central topic in ecology of microbiology.^[^ ^1^, ^2^ ^]^ The development of culture‐independent sequencing technologies has substantially expanded our characterization of microbial diversity,^[^ ^3^, ^4^ ^]^ which is restricted in its geographic and ecological distributions,^[^ ^5^, ^6^ ^]^ like plant and animal. For decades, ecologists have tried to understand which ecological and phylogenetic attributes lead to wider distribution of some taxa than others.^[^ ^7^ ^]^ It has been shown that sets of functional traits (e.g., specific leaf area and water‐use efficiency) that associate with major ecological roles could affect species’ niche breadth (i.e., the range of resources and conditions within which a species can survive).^[^ ^7^, ^8^ ^]^ However, a general understanding on how a multitude of species traits jointly influence niche breadth remains poorly understood for microorganisms, largely due to the technical challenges associated with characterizing their traits and diversity.^[^ ^9^, ^10^, ^11^ ^]^

**Figure 1:**
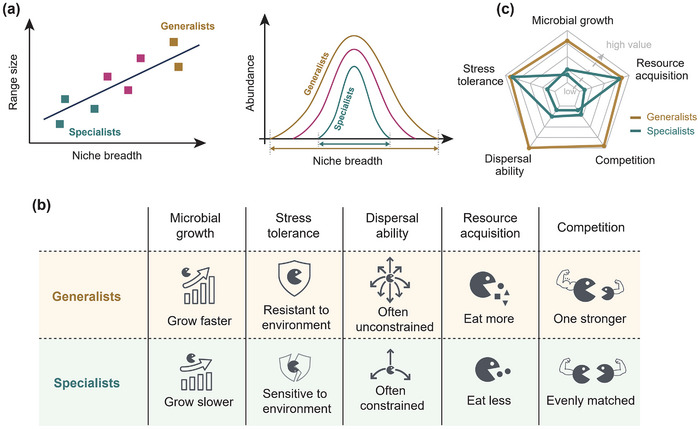
Conceptual framework for niche breadth based on life history strategies inferred from metabolic pathways and functional gene. a) Niche breadth is essentially the range of conditions under which an organism can live and is basic property of species. Each species has its own unique niche breadth. Generalists can survive in various environments and tend to have wider niche breadth, whereas specialists are restricted to a specific environment and tend to have narrower niche breadth. b) We hypothesize that bacterial niche breadth varying among species are related to their genome size, gene functional diversity, and life history strategies associated with growth, competition, stress tolerance, resource acquisition, and dispersal ability. c) We expect that bacterial taxa with a wide niche breadth typically exhibit a diverse range of traits associated with different life history strategies. These traits may include higher growth rates, competitive ability with other microbes, frequent dispersal, the ability to feed on a wide variety of substrates, and resilience to stressful abiotic conditions. In contrast, bacterial taxa with a narrow niche breadth are likely to possess a limited number of traits, mostly associated with a single or few life history strategy aspects.

Emerging knowledge of microbial physiology or life‐history traits offers a remarkable opportunity to expand trait‐based understanding of niche breadth through massive amounts of functional trait information inferred from genomes,^[^ ^12^ ^]^ such as the ability to either synthesize molecules with the utilization of energy, or break down complex substrates for resource acquisition. Besides, the spatial and ecological distribution of microorganisms is a concrete characterization of niche space in nature across the full n‐dimensional niche space, where each dimension is spanned by variables representing environmental conditions and resource availability (e.g., light and nutrients) (**Figure** **1**a).^[^ ^13^, ^14^ ^]^ For example, taxa with narrow niche breadth use a very specific set of resources and conditions, whereas taxa with wide niche breadth take advantage of general resources and large‐scale ecological space.^[^ ^7^ ^]^ Thus, this full dimensionality of niche space provides a framework for evaluating diverse microbial ecological or physiological strategies and enable us to directly link microbial performance to environmental regimes.

How do life‐history strategies affect microbial niche breadth along environmental gradient? The distributions of organisms across space and time represent their resource requirements and tolerances along abiotic and biotic axes in nature.^[^ ^15^ ^]^ The genomic information serves as the foundational material for understanding physiology and phenotype.^[^ ^16^ ^]^ It encompasses the metabolic mechanisms of primary and specialized metabolism, which play a crucial role in shaping the niche breadth through evolutionary and ecological processes. Although genomic studies are now prevailing,^[^ ^13^, ^17^ ^]^ there is still a limited understanding of genomic traits underlying life history strategies and niche breadth. This knowledge gap is crucial for comprehending microbial fitness in a specific environment and their impact on ecosystem functioning,^[^ ^18^ ^]^ such as soil carbon cycling and phytoremediation.^[^ ^12^, ^19^ ^]^ It is also vital for predicting the consequences of anthropogenic environmental change and climate change feedbacks on ecosystems.^[^ ^20^, ^21^ ^]^ One challenge to unraveling full multi‐dimension of trait space is enormous and complex uncharacterized metabolic diversity inferred from genomes, which hinders progress to place the vast diversity of the microbial genome into a coherent ecological context. Several conceptual theories have been proposed to characterize the microbial life history strategies.^[^ ^12^, ^18^, ^22^ ^]^ For example, the competitor‐stress tolerator‐ruderal (C‐S‐R) and high yield‐resource acquisition‐stress tolerance Y‐A‐S strategies map a standardized trade‐off set of phenotypic and physiological characteristics of microbial adaptation and function.^[^ ^12^, ^23^ ^]^ These frameworks provide effective approaches to collapse complex microbial genomes into ecological modules that facilitate the exploration of general patterns. Nevertheless, they do not clearly capture the life history strategies linked to microbial niche breadth, particularly traits involving dispersal capacity.

To better explain microbial niche breadth, we propose a new life history framework that expands the original CSR schema^[^ ^12^, ^23^ ^]^ to include five major groups: growth, competition, stress tolerance, resource acquisition, and dispersal ability (Table S1, Supporting Information). First, microbial growth strategies focus on investment in biosynthetic processes involved with central carbon metabolism and ATP synthesis. Second, if species could continue to survive or persist across various complex environments, they need to overcome variable resource availability and distribution (quantity and quality), stressful abiotic conditions including extremes of moisture, temperature, pH and salinity, and competition from other organisms. Thus, stress tolerance traits mainly include damage repair, osmolyte production, protection from desiccation and maintenance of cell integrity that help cope with abiotic stress. Third, traits of resource acquisition strategy involve in degradation of complex substrates, uptake of simple substrates as well as ability of autotrophy. Fourth, competitive strategy relates to secretion systems and antibiotic production that affect microbial interactions. Finally, greater dispersal ability is also required for broad distribution of generalists, which is largely related to cell motility and chemotaxis like flagellar assembly. Moreover, encoding traits for all these aspects of life history strategy involves a larger genome and more diverse functional genes.^[^ ^17^, ^24^ ^]^ We hypothesize that the specialist‐generalist continuum across niche breadth is related to their genome size, gene functional diversity, and life history strategies associated with growth, competition, stress tolerance, resource acquisition, and dispersal ability (Figure 1b). We expect that specialists with narrow niche breadth harbor a small number of traits associated with one life history strategy (small genome, dominated by genes related to one or a few aspects of life‐history strategies), whereas generalists with wide niche breadth will maximize a diversity of traits associated with different life history strategies (large genomes carrying traits associated with different life history aspects) (Figure 1c). By incorporating additional dimensions, the new life history framework provides a more comprehensive understanding of microbial niche breadth and the adaptations that enable organisms to occupy diverse niches. This framework can be used to investigate the distribution patterns of microorganisms across various ecological contexts.

To precisely characterize the niche breadth of microbial taxa, we collected 893 soil samples across China that span a wide range of environmental gradient at nation‐scale, covering different ecosystems, including forest, grassland and wetland. To determine the genomic features associated with niche breadth, the representative sequences of those identified bacterial taxa were matched to those with full genomes in GTDB database.^[^ ^25^ ^]^ First, we explored the taxonomic and phylogenetic patterns of bacteria with different niche breadths (calculated as all environmental variable taken together), and then identified life‐history strategies represented by metabolic modules and functional genes that are associated with bacterial niche breadth across specific environmental gradient (soil pH, nutrient dependence, temperature and moisture pressure). Finally, we evaluated the trade‐off among different life‐history strategies that determine niche breadth to reveal whether different life‐history strategies can co‐occur (**Figure** **1**).

## 2 Results

### 2.1 Niche Breadth Distribution across Taxonomic and Phylogenetic Profiles

To evaluate the niche breadth of bacterial phylotypes, we calculated the environmental range of each taxon under eight soil and climate conditions (Figures S1b and S2, Supporting Information), including mean annual temperature (MAT), mean annual precipitation (MAP), soil pH, organic matter (SOM), available phosphorus (AP), nitrate (NO_3_^‐^) and ammonium nitrogen (NH_4_^+^) and soil moisture. Six of these environmental variables were closely related to bacterial community composition, with Mantel ρ values ranging from 0.127 to 0.317 (Figure S3, Supporting Information). Before calculating the environmental range, we standardize the values of each environmental variable measured to have the same scale from 0 to 1. For each environmental variable, environmental range of each taxon was defined as the environmental breadth of soils where the taxon is present. Then the environment ranges for the eight environmental variables were averaged and summarized into a single index, representing the niche breadth of the given taxon.^[^ ^26^ ^]^

**Figure 2:**
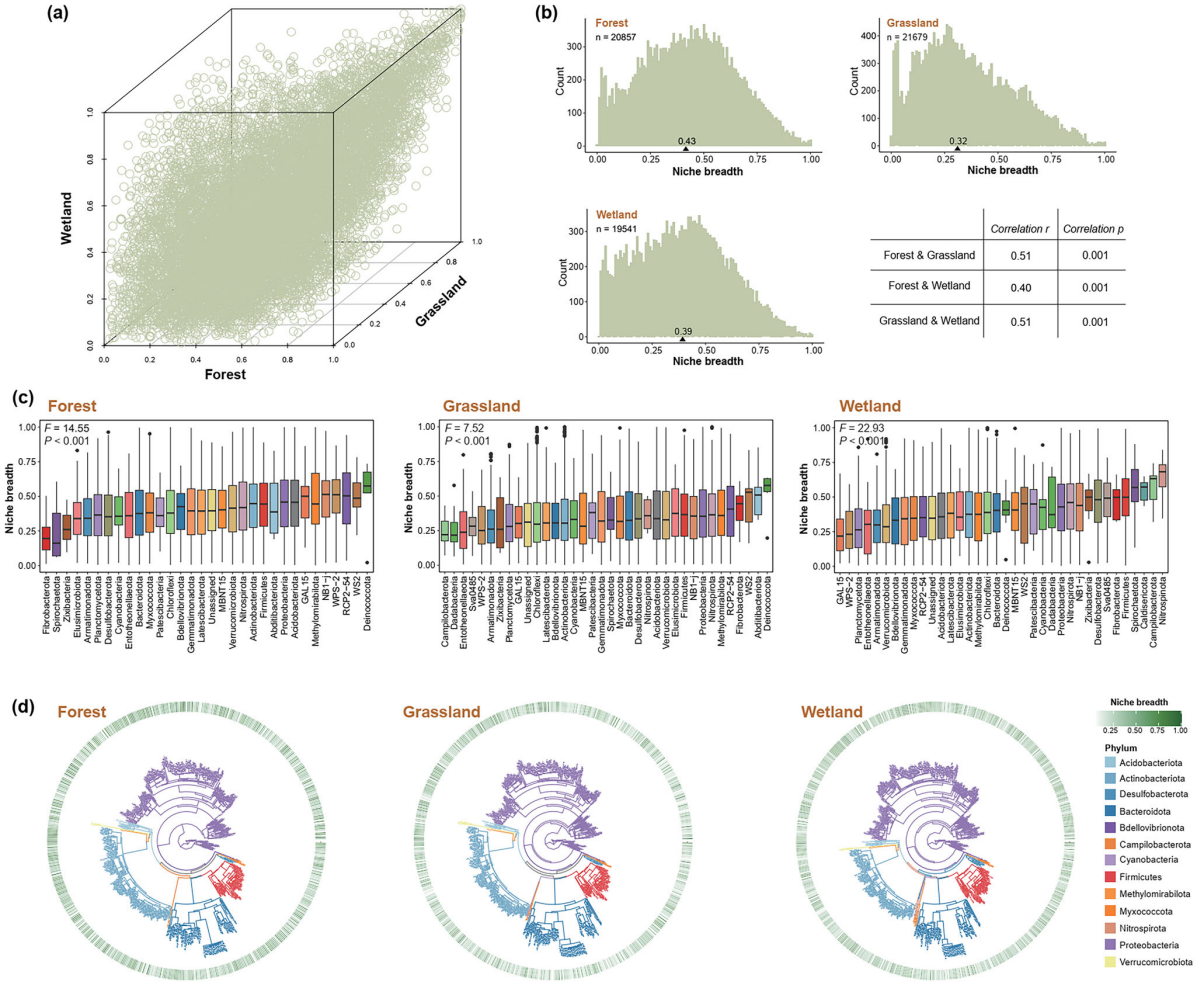
The relationships between niche breadth of bacterial taxa and taxonomic assignment and phylogenetic distribution in each ecosystem. a) Overall distribution of niche breadth of bacterial taxa in forest, grassland and wetland ecosystems. b) Distribution of niche breadth of bacterial taxa in each ecosystem. The triangle and the values above represent the median niche breadth. c) Box plots illustrating niche breadth distributions for bacterial taxa ranked by phylum in each ecosystem. The middle line represents the median; box edges denote the first and third quartiles; whiskers, 1.5 IQR (inter‐quartile range); points, outlier points. Niche breadth varies significantly between phyla (one‐way ANOVA). d) Panels show the phylogenetic trees of the ASVs which representative genomes was available in each ecosystem. The ASVs that match 100% identity and 100% coverage to the genome are displayed. Clades are colored by the affiliation at the phylum level. The outer ring depicts the bacterial niche breadth of the ASVs. The minimum niche breadth is depicted in white, and the maximum is depicted in green according to the legend.

To investigate the distribution of niche breadth of bacterial taxa throughout the prokaryotic tree of life, we identified 30,217 amplicon sequence variants (ASVs) using 16S rRNA gene sequence data from a total of 893 soil samples spanned a wide range of soil and climatic conditions at national scale, including 332 samples in forests, 277 samples in grasslands, and 284 samples in wetlands. These phylotypes covered a broad phylogenetic distribution, and belonged 799 genera, 363 family, 261 order, 123 classes and 53 phyla. The distribution of niche breadth value differs substantially across phylotypes (**Figure** **2**a,b). The median value of bacterial niche breadth in forests was the highest at 0.43, followed by 0.39 in wetlands and 0.32 in grasslands. Moreover, the shared taxa within three ecosystems have similar niche breadths across ecosystems (Figure 2b), implying a relatively stable property of niche breadth for bacterial taxa across different habitats. For example, taxa with broad niches in forest soils also exhibited wider niches in other two ecosystems.

We tested whether niche breadth differed across taxonomic groups. The bacterial niche breadth varies significantly across phyla in forests (one‐way ANOVA; *F*_31, 20789_ = 14.55, *P* < 0.001), grasslands (one‐way ANOVA; *F*_35, 21619_ = 7.52, *P* < 0.001) and wetlands (one‐way ANOVA; *F*_35, 19473_ = 22.93, *P* < 0.001; Figure 2c). Whereas, phylotypes within a given phylum have wide range of niche breadth, especially for dominant phyla such as Proteobacteria (ranging from 0.006 to 1), Actinobacteriota (ranging from 0.007 to 1) and Acidobacteriota (ranging from 0.004 to 0.999) in forest soils. We then assessed the phylum composition associated with different interval of niche breadth, and observed that the phylotypes from the same phylum distributed within different ranges of niche breadth (Figure S4a, Supporting Information), implying that phylum‐level information provided little insight for explaining bacterial niche breadth. Similar patterns were also found at finer Class and Order levels (Figure S4b,c, Supporting Information).

We further examined the association of phylogenetic information with niche breadth to test whether bacterial niche breadth is phylogenetically conserved. Actually, there was a weak phylogenetic signal across niche breadth, indicating that bacterial niche breadth was randomly distributed across the phylogenetic tree (taxa that are phylogenetically closely related would vary greatly in niche breadth; Figure 2d and Table S2, Supporting Information). For specific environmental variables, significant phylogenetic signal was only observed for pH, MAT and MAP in forests and NH_4_ in wetlands (Table S2, Supporting Information). These observations were also confirmed by weak relationship between phylogenetic distance and pairwise differences in niche breadth for all ASVs (Figure S5, Supporting Information). Overall, our results indicated that neither taxonomic nor phylogenetic information was a good indicator in explaining bacterial niche breadth.

### 2.2 Relationships Between Niche Breadth and Genomic Structure

The representative genomes in the GTDB dataset were obtained by matching the reference ASVs sequences, and were translated coding sequences for the functional annotation using MicrobeAnnotator.^[^ ^27^ ^]^ Overall, we obtained 1,657, 1,775 and 1,894 representative genomes in forests, grasslands, and wetlands, respectively. These representative genomes covered 31 phyla (Figure S6, Supporting Information), which represents a wide of taxonomic and phylogenetic distributions. Although these ASVs with the available genomes only are a relatively small fraction (7.94% to 9.69%) of total ASVs identified in our samples, these ASVs spanned the whole niche breadth range as the overall community (Figure S7, Supporting Information), suggesting that these genomes could be used to examine trait‐based mechanisms of niche breadth without preference biases.

**Figure 3:**
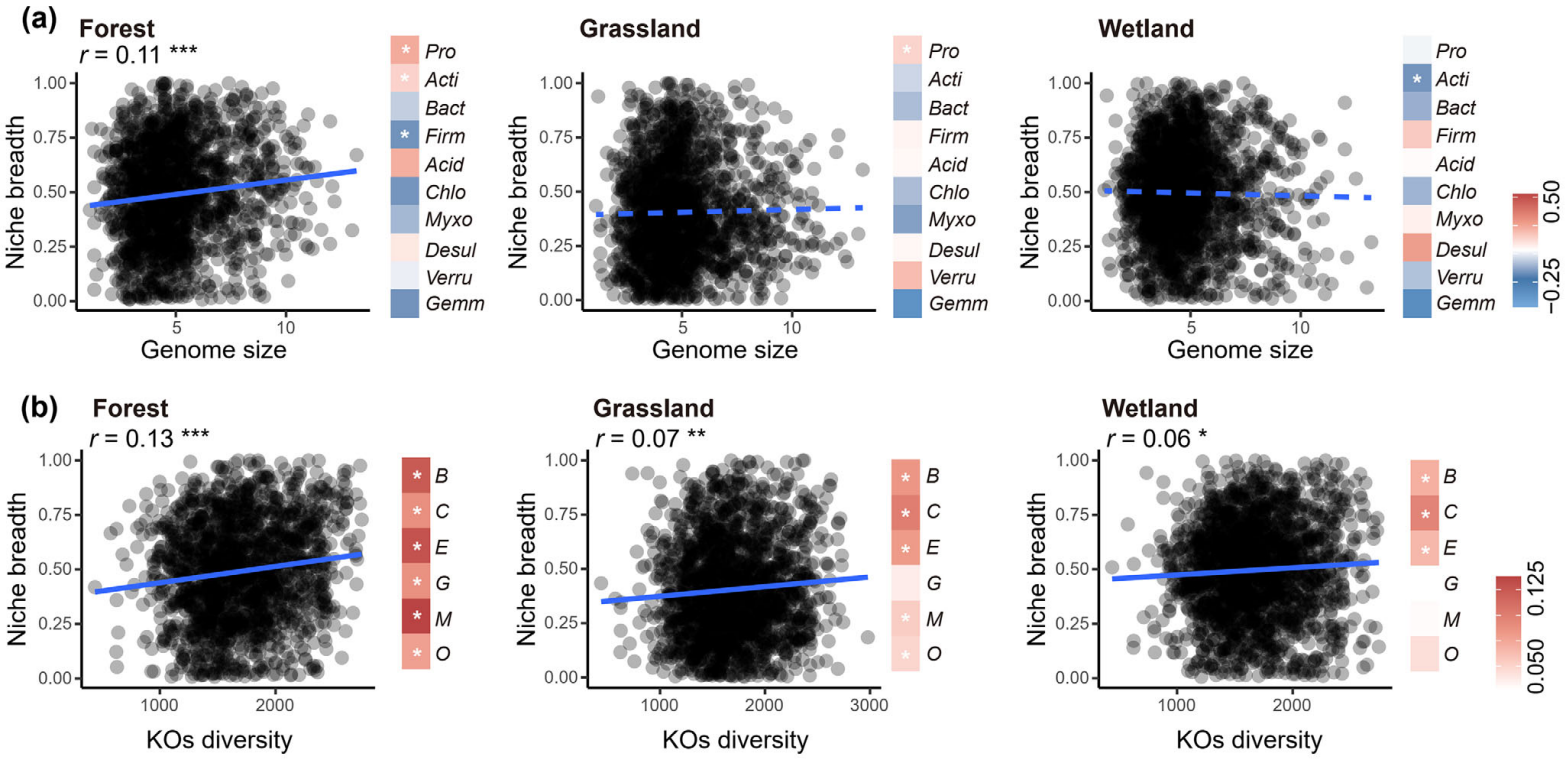
The genome size and functional diversity along niche breadth. a) The linear relationships between genome size and bacterial niche breadth was tested by Pearson correlation. Heatmap shows relationships between genome size and niche breadth at phylum‐level. Pro, Proteobacteria; Acti, Actinobacteriota; Bact, Bacteroidota; Firm, Firmicutes; Acid, Acidobacteriota; Chlo, Chloroflexi; Myxo, Myxococcota; Desul, Desulfobacterota; Verru, Verrucomicrobiota; Gemm, Gemmatimonadota. b) The linear relationships between functional diversity (S.KO) and bacterial niche breadth. Heatmap shows relationships between functional diversity and niche breadth at KEGG level 1 categories. B, Brite Hierarchies; C, Cellular Processes; E, Environmental Information Processing; G, Genetic Information Processing; M, Metabolism; O, Organismal Systems. Solid and dashed lines represent significant and non‐significant relationships, respectively. Asterisks in heatmaps indicate significant relationships. *P < 0.05, **P < 0.01, ***P < 0.001.

We then examined the relationships between genomic structure and niche breadth (**Figure** **3**). The bacterial niche breadth was positively correlated with genome size in only forest soils (Pearson’*r* = 0.11, *p* < 0.001; Figure 3a), and a relationship also observed within Proteobacteria and Actinobacteria. The bacterial niche breadth was not associated with guanine‐cytosine (GC) content and the coding DNA sequence (CDS). The bacterial KO diversity was positively correlated with niche breadth in forest soils (Pearson’*r* = 0.13, *p* < 0.001), grassland soils (Pearson’*r* = 0.07, *p* < 0.01) and wetland soils (Pearson’*r* = 0.06, *p* < 0.05). Positive correlations were also found with the KO diversity within the different metabolic pathway (KEGG level 1 categories) (Figure 3b).

### 2.3 Metabolic Modules and Functional Genes Associated with Niche Breadth

**Figure 4:**
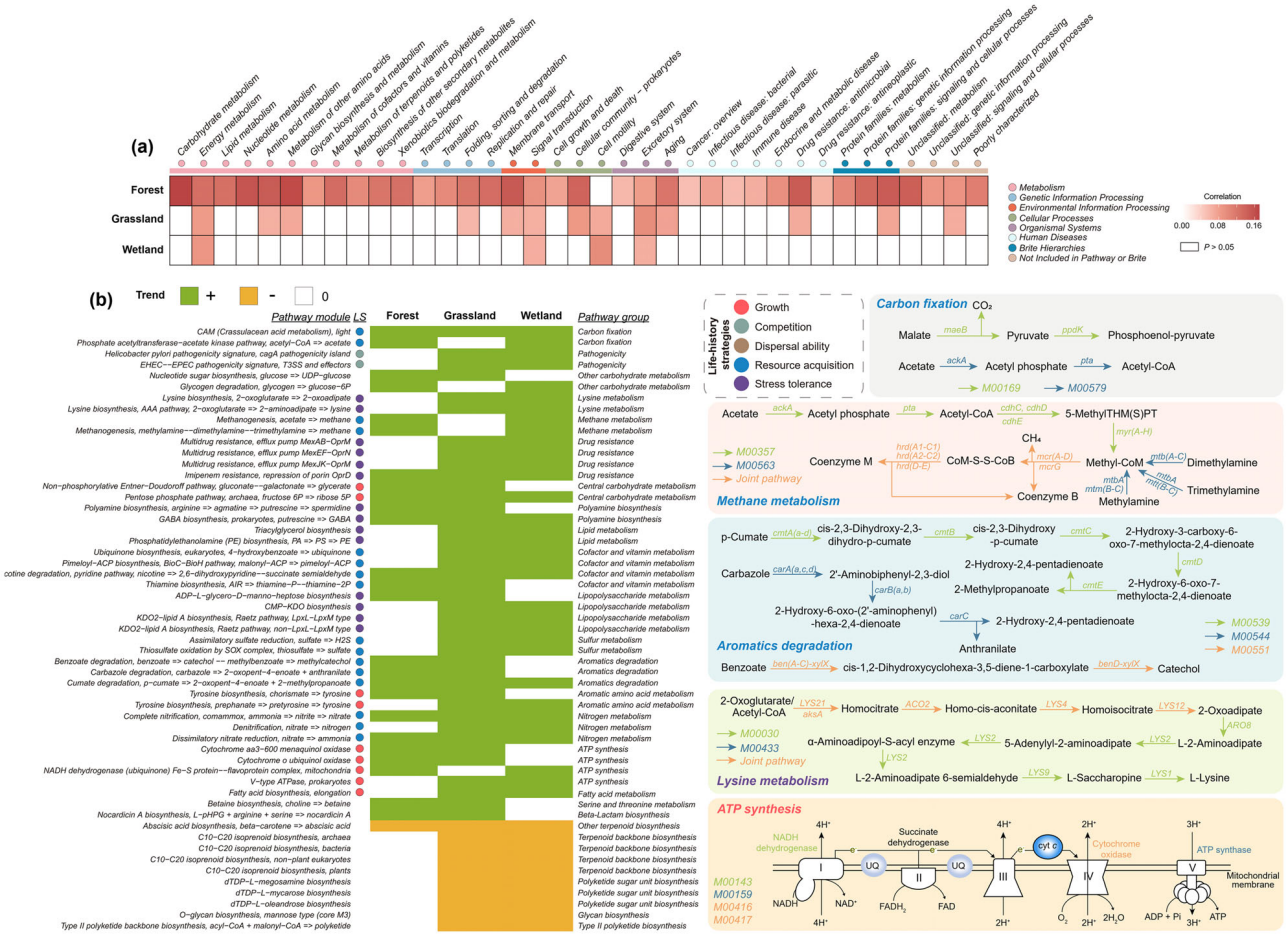
The relationships between metabolic pathways and bacterial niche breadth across ecosystems. a) Pearson's correlations between the total number of gene copies belonging to each category at Level 2 of KEGG categories and bacterial niche breadth in three ecosystems (forest, grassland, and wetland soils). Only functional categories that are significantly associated with niche breadth in at least one ecosystem are shown. b) Positive trends (green) indicate that the completeness of metabolic pathways is associated with wider niche breadth, suggesting that more complete or diverse metabolic pathways enable bacteria to occupy broader ecological niches. Negative trends (yellow) indicate that the completeness of metabolic pathways is associated with narrower niche breadth, which may reflect specialization in particular ecological niches. We only included metabolic pathways that showed shared relationships with niche breadth in at least two ecosystems, allowing us to identify common trends across ecosystems. Metabolic pathways are grouped according to the five life‐history strategies (resource acquisition, growth, stress tolerance, competition, and others), with each group highlighted in a distinct color to reflect their ecological role. Several representative metabolic pathways are visualized, e.g., carbon fixation (M00169 and M00579), methane metabolism (M00357 and M00563), aromatics degradation (M00539, M00544 and M00551), lysine metabolism (M00030 and M00433) and ATP synthesis (M00143, M00159, M00416 and M00417). These pathways were selected as representative examples of the key metabolic functions linked to bacterial life‐history strategies. LS, Life‐history strategies.

A set of KEGG modules (in forests: PERMANOVA *R^2^* = 0.0029, *p* < 0.001; in grasslands: PERMANOVA *R^2^* = 0.0022, *p* < 0.001; in wetlands: PERMANOVA *R^2^* = 0.0034, *p* < 0.001) and KOs (in forests: PERMANOVA *R^2^* = 0.0020, *p* < 0.001; in grasslands: PERMANOVA *R^2^* = 0.0028, *p* < 0.001; in wetlands: PERMANOVA *R^2^* = 0.0033, *p* < 0.001) exhibited significant variations among bacterial niche breadths (Figure S8, Supporting Information). Multiple KEGG pathways were positively correlated with niche breadth, particularly in forest soils where most of the pathways were closely associated with niche breadth (**Figure** **4**a; Figure S9, Supporting Information). These results highlighted the tight associations between genomic traits and bacterial niche breadth.

Specifically, we identified 75, 65, and 74 pathways that had the significant association with bacterial niche breadth in forests, grasslands and wetlands, respectively. Specifically, 55 of those pathways had the consistent association with niche breadth in at least two ecosystems (Figure 4b), among which 45 pathways were positively associated with bacterial niche breadth (Figure 4b). These modules mainly involved in resource acquisition and environmental adaptation such as degradation of complex substrates, chemoautotrophy and amino acids, lysine, polyamine related to stress tolerance and maintenance of cellular integrity (Figure 4b). Fewer metabolic pathways and genes were enriched in taxa with narrow niche breadth than that with broad niche breadth. These enriched functions include terpenoid backbone biosynthesis and polyketide sugar unit biosynthesis (Figure 4b). For KO genes, we found that the presence of a set of specific functional gene categories was significantly correlated with different range of niche breadth (Figure S11, Supporting Information). Although some genes were negatively correlated with niche breadth, the presence of most genes led to a wider niche range of those taxa. A total of 1421 KOs were identified that had the significant association with bacterial niche breadth in at least two ecosystems, and these KOs belonged to 66 third‐level KEGG categories (Figure S12, Supporting Information). For example, all 32 KOs involved in secretion system and 24 KOs involved with flagellar assembly were more common in those taxa with large niche breadth. We also identified module pathways that had the significant association with bacterial niche breadth of different environmental factors (Figure S10, Supporting Information). The niche breadth of MAT was largely related to ATP synthesis, aromatic amino acid metabolism and aromatics degradation and niche breadth of soil moisture was mainly associated with lysine and nitrogen metabolism. Modules for cofactor and vitamin metabolism, drug resistance, lipid, lipopolysaccharide and lysine metabolism had higher completeness levels in taxa with wide nutrient niche. Overall, our analysis showed that functional modules and genes associated with niche breadth fit in the different microbial life history strategies.

In general, these functional modules and genes could be assigned to five microbial strategies involved with microbial growth, competition, stress tolerance, resource acquisition, and dispersal ability (Figure 4b; Figure S12, Supporting Information). For microbial growth, the identified modules of central carbohydrate metabolism and ATP synthesis, and genes for propanoate, butanoate, pentose phosphate, pyruvate metabolism were positively associated with bacterial niche breadth. With regard to competition, modules assigned to pathogenicity and genes assigned to secretion system and prokaryotic defense system had higher completeness levels and overrepresented in taxa with wide niche. Resource acquisition associated modules and genes of chemoautotrophy (carbon fixation, methane metabolism, sulfur metabolism and nitrogen metabolism), degradation of complex substrates (cofactor and vitamin metabolism, aromatics degradation) and uptake of simple substrates (transporters and ABC transporters) had higher completeness levels in taxa with wider niche. Modules and genes associated with stress tolerance were also positively correlated with bacterial niche breadth, such as osmolyte production (lysine biosynthesis, polyamine biosynthesis in modules), biomolecular damage repair (replication and repair, DNA repair and recombination proteins in level 3 of KEGG categories), maintenance of cellular integrity (lipopolysaccharide biosynthesis, lipid biosynthesis in modules) and biofilms (biofilm formation in level 3 of KEGG categories). Finally, genes for flagellar assembly known to be involved in dispersal ability overrepresented in taxa with wide niche breadth.

### 2.4 Specialist‐Generalist Continuum Varying Across Life History Strategies

**Figure 5:**
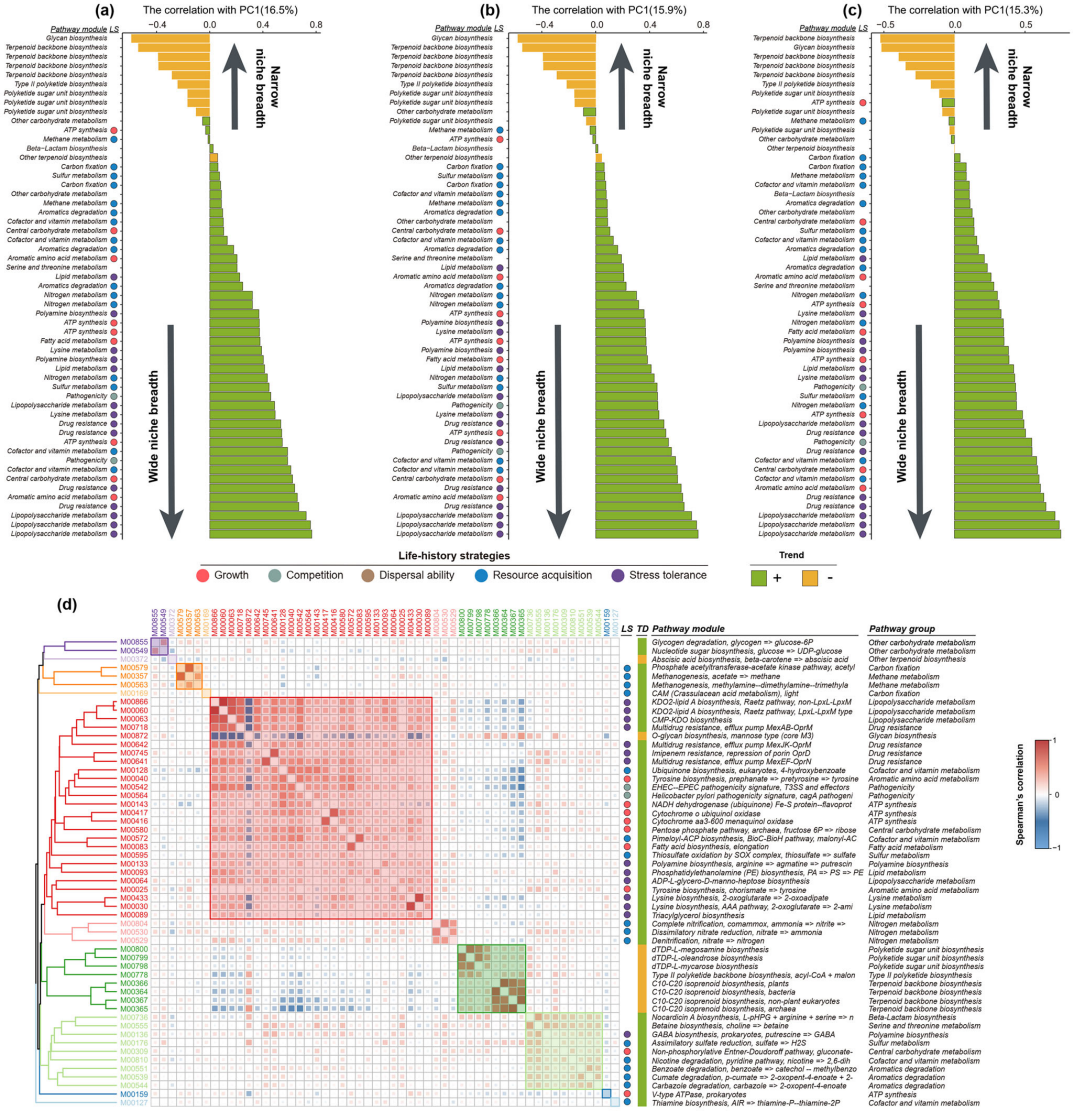
Trait correlations and functional clusters. a–c) Bars display the correlation (PC loadings) of individual metabolic modules with PC1 (principal component axis 1) in forest a), grassland b) and wetland soils c). PC1 captures the wide‐narrow continuum of niche breadth across the 55 metabolic modules, reflecting the variation in ecological strategies. Green bar indicate that the completeness of metabolic pathways is associated with wider niche breadth, and yellow bar indicate that the completeness of metabolic pathways is associated with narrower niche breadth. These associations highlight the potential ecological trade‐offs between generalist and specialist microbial taxa. d) Correlation coefficients for pairwise Spearman correlations among 55 metabolic modules that had shared relationships to niche breadth in at least two ecosystems (Figure 4b) in bacterial taxa. Traits are ordered based on the similarity of their correlation patterns, as determined by hierarchical clustering (Ward's method), with colors separating distinct clusters identified in the analysis (maximal silhouette width: K = 10). The size of the square denotes the relative strength of the correlation, with red denoting positive correlations and blue denoting negative correlations. Larger squares represent stronger correlations, whereas smaller squares indicate weaker relationships. LS, Life‐history strategies; TD, trends with the completeness of metabolic pathways.

It is generally known that a fundamental trade‐off exists among life history strategies.^[^ ^28^ ^]^ To elucidate the trait associations that underlie bacterial life‐history strategies, we quantified the full dimensionality of metabolic modules via principal component analysis (PCA; Figure S13, Supporting Information). The first principal component (PC1) explained 16.5% of the total variance in forests, 15.9% in grasslands, and 15.3% in wetlands, highlighting the major metabolic gradients in microbial life history strategies. The correlation (PC loadings) of metabolic modules with the first PC axis unveiled an overarching trait continuum, spanning from modules exhibiting negative associations with niche breadth to those displaying positive associations with niche breadth (**Figure** **5**a–c).

We subsequently identified groups of module pathways that construct tightly coupled clusters and reflect distinct aspects of microbial life history. The results showed that these 55 module pathways can be grouped into six trait clusters. Among these, four clusters, namely the red, shallow green, orange, and pink clusters, exhibited positive associations with bacterial niche breadth. Interestingly, module pathways from the same microbial life history category were captured by distinct clusters. For instance, the largest cluster (Figure 5d, red cluster) encompassed life history strategies related to microbial growth (central carbohydrate metabolism and ATP synthesis), competition (pathogenicity), stress tolerance (lipopolysaccharide, lipid, and lysine biosynthesis), and resource acquisition (cofactor and vitamin metabolism, and sulfur metabolism); stress tolerance (polyamine biosynthesis) and resource acquisition (aromatics degradation) formed second largest cluster (Figure 5d, shallow green cluster). These observations suggest that a broad‐niche taxon may possess multiple facets of life history strategies necessary to support a wider niche breadth simultaneously in a given environment and snapshot. Moreover, the biological significance of the identified clusters lies in their reflection of key ecological functions: growth‐related pathways support metabolic demands for rapid growth, competitive pathways facilitate survival in resource‐limited environments, stress tolerance pathways confer resilience under harsh conditions, and resource acquisition pathways enable efficient utilization of available resources. Together, these findings provide valuable insights into the metabolic underpinnings of microbial adaptation to diverse ecological niches.

**Figure 6:**
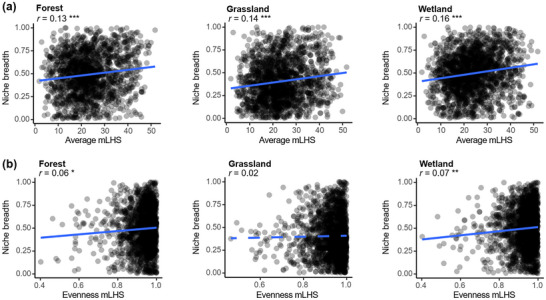
The relationships between the average completeness and Pielou evenness of modules aggregated within Life History Strategies (mLHS) and niche breadth. a) Associations between the average mLHS and bacterial niche breadth in three ecosystems. The linear relationships were tested by Pearson correlation. b) Associations between the evenness mLHS and bacterial niche breadth in three ecosystems. The evenness mLHS was calculated as the Pielou evenness of the mean completeness of modules belonging to life history strategies. The higher the Pielou evenness, the closer each life history strategy is to completeness, indicating that the taxon maximizes a diversity of traits associated with different life history strategies. *P < 0.05, **P < 0.01, ***P < 0.001.

To further test whether the specialist‐generalist continuum varies across life history strategies, we examined the relationships between bacterial niche breadth and the average/evenness of **m** odules aggregated within **L** ife **H** istory **S** trategies (mLHS). As expected, the average mLHS was positively correlated with niche breadth in forest soils (Pearson’*r* = 0.13, *p* < 0.001), grassland soils (Pearson’*r* = 0.14, *p* < 0.001) and wetland soils (Pearson’*r* = 0.16, *p* < 0.001) (**Figure** **6**a). The separated four life‐strategies (resource acquisition, growth, stress tolerance, and competition) was positively correlated with niche breadth in all three ecosystems (Figure S14, Supporting Information). The evenness mLHS was positively correlated with niche breadth in forest soils (Pearson’*r* = 0.06, *p* < 0.05) and wetland soils (Pearson’*r* = 0.07, *p* < 0.01) (Figure 6b). These findings support our hypothesis that bacterial taxa with wider niche breadth maximize a diversity of traits associated with different life history strategies, whereas specialists tend to harbor a smaller number of traits associated with fewer life history strategies.

### 2.5 Functional Potential of Taxa Occupying Different Niche Breadths

To investigate the functionality of bacterial taxa occupying different niche breadths, we examined the distribution of bacterial functional traits associated with the biogeochemical cycle across a gradient of niche breadth. First, we found that bacterial niche breadth was significantly associated with a number of microbial functional groups important for C decomposition. Most of the detected C decomposition genes, involving in degrading starch, hemicellulose, cellulose, chitin, aromatics and lignin, were enriched in broad‐niche taxa in forest soils (Figure S15a,b, Supporting Information). In grassland and wetland soils, the genes involved in labile C degradation, e.g., starch, hemicellulose and cellulose, were decreased with niche breadth, while genes associated with recalcitrant C degradation increased with niche breadth (Figure S15a,b, Supporting Information).

In addition, bacterial niche breadth was related with genes involved in N cycling, microbial phosphorus utilization and sulfur metabolism (Figure S15c–e, Supporting Information). For example, the key genes (for example, *hao*, *narG*, *norB* and *nosZ*) in nitrification and denitrification processes were overrepresented in taxa with wider niche breadth in grassland and wetland soils (Figure S15c, Supporting Information). Microbial phosphorus utilization genes, such as *gcd* for inorganic P solubilization genes, *phnN* and *phnH* for organic P mineralization, *pstS*, *phnE* and *ugpE* for P transportation and *phoB* for P regulation, were enriched with bacterial niche breadth increasing in two out of three ecosystems (Figure S15d, Supporting Information). Furthermore, we identified genes for dissimilatory sulfate reduction (for example, *aprA*, *aprB*, *dsrA* and *dsrB*) as overrepresented in taxa with higher breadth niche in wetland soils (Figure S15e, Supporting Information). Overall, we revealed that the genes involved in biogeochemical cycling enriched in taxa with higher niche breadth, indicating the potential major roles of the bacterial generalists in nutrient cycling processes.

## 3 Discussion

Niche breadth represents the range of condition where an organism is observed, and is intricately linked to evolutionary, ecological, and life history attributes of organisms.^[^ ^29^ ^]^ Revealing why niche breadth vary across species and what determines niche breadth is essential for predicting species distribution patterns and their impact on ecosystem functions in response to environmental changes.^[^ ^30^ ^]^ Here, our study described the relationships between niche breadth and genomic life history trait of soil bacteria, providing new insights for future trait‐based research of soil bacteria biogeography.

In the present study, we collected over 2,000 bacterial genomic datasets, and identified a set of metabolic pathways and genes representing traits consistently linked to niche breadth across ecosystems (Figure 4; Figure S12, Supporting Information). Most of the identified traits related to niche breadth were associated with five life history strategies, including microbial growth, competition, stress tolerance, resource acquisition, and dispersal ability, which capture key traits for microorganisms to grow and survive in specific environment. Focusing on organismal traits enables us to simplify intricate biological communities into ecologically cohesive units, thereby facilitating the identification of adaptive mechanisms essential for survival in diverse environments. Recent researches had indicated that genome size and functional content of the genomes were linked to bacterial ubiquity and adaptation to different environmental conditions.^[^ ^31^ ^]^ Genome size reflect largely the number of different coding genes, which is believed to be associated with versatility with signal transduction.^[^ ^24^ ^]^ This is consistent with our findings that gene functional diversity is positively related with bacterial niche breadth. In addition, the necessary link between genome size and more complex metabolism and resource acquisition strategies suggests that an expansion of metabolic capabilities could help bacteria possess a large niche breadth.^[^ ^17^ ^]^ Although previous evidence has shown that specific traits could affect niche breadth or range size,^[^ ^10^, ^32^ ^]^ few studies have systematically incorporated these traits into the framework of life history strategies. This approach enhances the understanding of niche breadth driven by soil bacterial traits and provides support for improved niche breadth prediction models.

Although metabolic diversity is simplified by life history classification, there are many pathways and genes involved in multiple life history strategies, such as transporters allowing a direct uptake of substrates and cofactors and synthesized molecules involved in metabolic and energy processes.^[^ ^33^ ^]^ For example, we identified the pentose phosphate pathway (PPP) in both metabolic pathways and genes, which was positively associated with bacterial niche breadth across soils (Figure 4). The PPP is a fundamental component of cellular metabolism and involved in carbon homoeostasis as well as in promoting precursors for nucleotide and amino acid biosynthesis.^[^ ^34^ ^]^ Moreover, the PPP is considered as a major contributor to the intracellular survival of the bacterium by genetic and imaging approaches and constitutes a metabolic hub like crossroads of glycolysis, the tricarboxylic acid cycle and other pathways, such as fatty acid degradation and sulfur metabolism.^[^ ^34^, ^35^ ^]^ The PPP is thus regarded as intrinsic virulence attributes of pathogenic bacteria (e.g. *Francisella novicida*) and represent potential targets for antibacterial strategies.^[^ ^35^ ^]^ These results indicate that PPP is not only involved in microbial growth, but is also linked to bacterial pathogenicity, competitive ability, and stress tolerance. We also identified two pathogenicity pathways (M00542, T3SS and effectors; M00564, cagA pathogenicity island) that are consistently associated with bacterial niche breadth. These similar pathways, which do not belong to a single life history strategy, is also found in the ATP synthesis module. The identified Fe‐S protein/flavoprotein complex (M00143, NADH dehydrogenase) are most commonly known for their role in remaining the generation of ATP via mediating electron transfer within the mitochondrial respiratory chain.^[^ ^36^ ^]^ In addition, Fe–S clusters also have prominent functions in several diverse biological processes, including photosynthesis, nitrogen fixation,^[^ ^37^ ^]^ RNA modification, and DNA replication and repair and play an important role in the production of mitochondrial ROS.^[^ ^38^ ^]^ Therefore, although a general perspective of life history is attractive, these strategies do not map clearly to simple microbial systems, which requires a deeper understanding of the correlations and trade‐offs among life history strategies.

Our analysis differs from previous approaches that identify genomic features associated with adaptations to pH,^[^ ^39^ ^]^ and we focused on locating the genomic traits related to niche breadth from the completeness of metabolic pathway modules and the presence/absence functional genes. Indeed, the simplest traits are encoded by just one genetic locus, so the presence or absence of this gene determines a particular phenotype. In addition, most traits are much more complex and determined by more than one gene,^[^ ^40^ ^]^ such as ATP synthesis and polysaccharide degradation. The discovery of some genes in the pathway suggests that this pathway may exist, and therefore the completeness of pathway modules determines their phenotype. This approach allows us to thoroughly understand the associations between simple and complex traits and niche breadth, making up for the unexplored complex traits caused by focusing only on the presence or absence of genes. By including completeness of pathway modules (some genes in the pathway and all genes in the pathway), our approach better incorporates those traits involved with multiple genes, such as biosynthesis and degradation, which could not be realized at single gene level. For example, we identified triacylglycerol and phosphatidylethanolamine biosynthesis involved in lipid synthesis and benzoate, carbazole and cumate degradation engaged in aromatics degradation in the metabolic modules. These findings suggest that taxa with higher pathway completeness may have the potential to adapt to a broader niche breadth. Our study integrates pathway module into genomic trait, offering new perspectives to advance trait‐based understanding of niche breadth.

Although bacterial niche breadth cannot be well distinguished by taxonomic identity, we found that some bacterial phyla had broader niche breadth in both forest and grassland soils, such as Deinococcota and WS2 (Figure 2c). Deinococcota is a phylum of tenacious environmental bacteria that are a highly resistant to extraordinary amounts of both sustained and acute ionizing radiation as well as desiccation. Some of these lineages were found at terrestrial hot springs,^[^ ^41^ ^]^ marine sediments^[^ ^42^ ^]^ and cold deserts in Antarctica,^[^ ^43^ ^]^ which represented widespread taxa in geochemical environments worldwide.^[^ ^44^ ^]^ Deinococcota possesses a complete set of DNA repair genes and powerful antioxidative mechanisms in stress tolerance and a capacity for atmospheric chemosynthesis and photoheterotrophy in resource acquisition, which allows for extreme tolerance to ionizing radiation and desiccation, causing wider niche breadth for Deinococcota.^[^ ^43^, ^45^ ^]^ Phylogenetic information for predicting niche breadth present considerable uncertainty, which is consistent with previous studies revealing weak associations between ecological patterns and phylogeny.^[^ ^32^ ^]^ Although microbial traits were proven to be phylogenetically conserved,^[^ ^40^, ^46^, ^47^ ^]^ directly establishing a link between phylogenetic information and ecological patterns is challenging, especially given that ecological patterns involve interactions of multiple traits.^[^ ^15^, ^39^, ^48^ ^]^ In the present study, the niche breadth of soil pH is more phylogenetically conserved than other environments (Table S2, Supporting Information). Soil pH preference appears to be relatively conservative in prokaryotic traits compared to moisture and temperature,^[^ ^40^ ^]^ in agreement with past studies showing that soil pH is the most dominant driver of biogeographical distribution of deep phylogenetic clades.^[^ ^49^, ^50^ ^]^ Since the evaluation of niche breadth took into account multiple environmental factors, including soil pH, moisture, temperature and nutrients, and is a comprehensive index, the phylogenetic range and taxonomic levels of conservation across different environmental conditions disperse the phylogenetic conservatism of niche breadth.^[^ ^51^ ^]^

Species with different niche breadths not only differ in life history strategies, but also have different functional roles in ecological processes.^[^ ^52^, ^53^ ^]^ We combined whole‐genome comparisons with niche breadth to show that generalists with broader niche breadth exhibit higher potential to participate in soil C turnover and nutrient cycling processes, providing important supporting evidence for the deploy and utilization of broad‐niche microorganisms. Managing species‐rich communities of soil microbiomes remains a major challenge, thus deterministically selection based on distribution range or niche breadth provide ecological insights into prioritizing keystone membership.^[^ ^54^ ^]^ Taken together, our results indicate that the niche breadth of species can be an important consideration in determining soil functions and processes and should be considered in asking certain key species that drive ecosystem function.

Although microbial traits can help reveal the underlying mechanisms of microbial biogeographic patterns, several limitations exist for characterizing microbial traits in our study. First, two individual strains classified within the same species may be of different ecotypes and perform different functions in the environment. The genome‐level diversity across ecologically distinct bacterial populations be responsible for niche differentiation and allow the discrimination of different ecotypes.^[^ ^55^ ^]^ For example, a global survey of *Curtobacterium* genus revealed an abundant OTU (99% identity) containing over 202 sequences isolated from all seven terrestrial ecosystems, and these isolates contained many diverse GH families.^[^ ^56^ ^]^ Second, our characterization of bacterial life‐history strategies remains poor, with is limited by the restricted number of reference genomes and gene functional characteristics.^[^ ^17^, ^57^ ^]^ Moreover, the reference database for annotated genomes is very limited for environment such as soil, which would reduce the annotation scope of genomic data and limit analysis conclusions. Finally, it should be noted that the weak correlations suggest that the relationship between mLHS and niche breadth, while statistically significant, may reflect only a subtle trend rather than a strong biological mechanism. Future studies should focus on a deeper exploration of microbial life history strategies by incorporating more detailed strategy information and utilizing genomes from experimentally isolated strains. By expanding the range of strategies considered and using strain‐based data, it can better capture the complexity of microbial adaptation to diverse environments and improve the interpretation of ecological trends.

## 4 Conclusions 

The mechanism of niche breadth of species is an important and fundamental ecological issue. The life history strategies of species determine their environmental adaptation and niche breadth, but these strategies remain poorly unclear. A major challenge is that how to relate the complexity and variability of metabolic traits, which shape species fitness, to the coherent macroecology context of niche breadth. Here, we employ taxonomically and phylogenetically diverse soil bacteria as model systems to uncover the key microbial traits that determine niche breadth. Importantly, we introduce a novel life history framework that expands upon the original CSR schema to encompass five major groups: growth, competition, stress tolerance, resource acquisition, and dispersal ability, and links these life history strategies to bacterial generalism. Overall, our study provides a baseline understanding of taxonomy, life‐history strategy and functional potential of soil bacteria along niche breadth in soils, which is especially important if we are to develop knowledge and integrated approaches aimed at conserving soil microbial diversity for healthy and functional soils.

## 5 Experimental Section

### Datasets and Sequence Processing

To obtain the niche breadth of bacteria taxa across a broad environmental gradient, 16S rRNA gene sequencing of soil datasets from distinct natural ecosystems (forests, grasslands and wetlands) across China was used. Soil samples were conducted along the 3400 km latitudinal gradient from 23°39’ N to 47°79’ N and the 3200 km longitudinal gradient from 86°30’E to 126°10’E, which that represented a broad range of environmental gradients in climate, soil and vegetation types (from tropical to boreal zones) (Figure S1a, Supporting Information).

Soil samples were collected in July and August 2019. At each sampling site, soil samples from three ecosystems were collected, with the maximum distance between them not exceeding 5 km. At each ecosystem, four to ten plots were established randomly. Soil samples were collected by taking three soil cores with a 5‐cm‐diameter auger to a depth of 20 cm in each plot. To avoid cross contamination across ecosystems, we thoroughly rinsed the soil auger using clean water after sampling, then applied a 75% alcohol solution to its surface and placed the auger bit into a sterile bag for safekeeping until the subsequent sampling event. A total of 893 soil samples were collected in three ecosystems. These soil samples were sieved through a 2.0‐mm mesh to remove plant roots, litter, rocks, and other debris. Each soil sample was divided into two subsamples where one set was frozen at −80 °C for DNA extraction and microbial analysis and the other set was air dried for measurement of soil physical and chemical properties.

The diversity of soil bacteria was measured by amplicon sequencing using an Illumina MiSeq platform. Genomic DNA was extracted from 0.5 g of the soil samples using the MP FastDNA spin kit for soil (MP Biomedicals, Solon, OH, USA) according to the manufacturer's instructions. A portion of the prokaryotic 16S rRNA gene (V4‐V5 region) was sequenced using the 515F (5’‐GTGYCAGCMGCCGCGGTA‐3’) and 907R (5’‐CCCCGYCAATTCMTTTRAGT‐3’) primer sets. Bioinformatics processing, including filtering, dereplication, sample inference, chimera identification, and merging of paired‐end reads, was performed using the Divisive Amplicon Denoising Algorithm 2 (DADA2), a model‐based approach for correcting Illumina amplicon errors without constructing OTUs.^[^ ^58^ ^]^ Compared to other methods, DADA2 identified more real variants and output fewer spurious sequences. The taxonomical annotation of the representative sequences of amplicon sequence variants (ASVs) was performed with a naïve Bayesian classifier using the Silva v. 138 database.^[^ ^59^, ^60^ ^]^ All bioinformatics processing was done in the dada2 package in R. Bacteria were defined as all prokaryotic taxa, except Archaea, chloroplasts, and mitochondria in 16S rRNA gene for subsequent analyses. Samples were rarefied to the same sequence depth (15 000 reads per sample). Overall, we obtained a total of 31 402 soil bacterial amplicon sequence variants (ASVs) in three ecosystems for further niche breadth analysis.

### Statistical Inference of Niche Breadth

It was selected eight environmental variables to represent niche breadth of soils, including mean annual temperature (MAT), mean annual precipitation (MAP), soil pH, organic matter (SOM), available phosphorus (AP), nitrate (NO_3_^‐^) and ammonium nitrogen (NH_4_^+^) and soil moisture. These climatic and soil factors cover temperature, moisture, nutrients, and pH of soil environment and are the most important predictors of bacterial diversity patterns at the continental and global scales.^[^ ^61^, ^62^ ^]^ Here, MAT and MAP with a spatial resolution of 2.5 min (≈4.5 km) from the WorldClim database was extracted (https://www.worldclim.org/). Soil pH was assessed in a 1:5 suspension (soil to distilled water) using a pH meter. Organic matter was determined calorimetrically following oxidation with a combination of potassium dichromate and sulfuric acid. Soil moisture was measured by the gravimetric method after samples were oven‐dried at 100 °C for 24 h. NO3‐N and NH4‐N concentrations were measured using 1M KCl solution with Continuous‐Flow AutoAnalyzer. Available phosphorus concentrations were extracted by NaHCO3 and measured by molybdenum blue colorimetry.

These eight environmental variables show broad gradients at our sampling sites and can well describe niche breadth. For example, MAP and MAT in these sites are from 99 to 1775 mm and −2.8 to 21.9 °C, respectively. Soil pH ranged from 4.63 to 10.18 and soil C ranged from 4.64 to 60.22 g kg^−1^ across all of the survey sites. For each environmental variable measured, standardize the values to have the same scale from 0 to 1. Environmental range was defined as the breadth of each environmental condition where a taxon is present. Therefore, before calculating the environmental range, we removed phylotypes that occurred only in single sample. Then the environment range normalized values of the eight environmental variables were averaged and summarized into a single index, representing multidimensional niche breadth of soil bacteria. Finally, the niche breadth of bacterial taxa based on their presence or absence in the soil samples was calculated. The taxonomic identity and the phylogenetic signals for bacterial niche breadth by calculating Blomberg's *K* was tested.^[^ ^63^ ^]^ The *K* value describes how well a taxon was correlated with phylogeny as expected in a Brownian motion‐based metric of the strength of phylogenetic signal.^[^ ^64^ ^]^ *K* values closer to zero indicate a random or convergent pattern of evolution, while *K* values greater than 1 indicate strong phylogenetic signals and conservatism of traits. Blomberg's *K* was calculated using the ‘multiPhylosignal’ function in the “picante” R package.^[^ ^65^ ^]^

Phylogenetic analysis was performed using the PHYLIP software package to infer evolutionary relationships among the bacterial species. The Seqboot program was first used to generate 1000 bootstrap replicates through resampling, providing a basis for assessing the robustness of the phylogenetic tree. For each replicate, a distance matrix was computed from the DNA sequences using the Dnadist program, with the F84 model selected for distance calculations due to its suitability for datasets with significant sequence variation. Subsequently, the Neighbor program was employed to construct phylogenetic trees using the Neighbor‐Joining (NJ) method. A consensus tree was generated using the Consense program, which integrated the topologies of all bootstrap replicates to produce a final tree reflecting the confidence of the inferred relationships. The resulting tree was output in Newick format for detailed exploration of phylogenetic relationships.

### Genome Search and Annotation

To obtain the genomic information of those ASVs to determine the relationship between genomic traits and niche breadth, the ASVs with bacterial niche breadth were matched to the annotated GTDB,^[^ ^25^ ^]^ using vsearch to identify those ASVs for which representative genomes was available. We acknowledge that the representative genomes identified using representative partial 16S rRNA gene sequences from ASVs were not necessarily identical to the genomes in situ, but this method provides us with a species‐perspective research framework using genomic datasets. This method was widely used in the study of microbial ecology.^[^ ^13^, ^39^, ^66^ ^]^ Matches were determined using BLASTn at ≥99% identity and ≥90% coverage. In situations where a single ASV matched multiple genomes with the same similarity, the most complete genome was selected. These genomes were carefully selected and classified as “isolation,” “high‐quality genome,” and “medium‐quality genome” based on GTDB metadata (Release 214). The proportion of these groups was as follows: 69.8% isolation genomes, 15.1% high‐quality genomes, and 15.1% medium‐quality genomes. Overall, a total of 1657, 1775 and 1894 representative genomes forests, grasslands, and wetlands, respectively, for subsequently functional annotation was matched. These ASVs with available genomes accounted for 7.94% for forests, 12.80% for grasslands and 9.69% for wetlands of the total ASVs, respectively.

The annotated genomes were then processed with MicrobeAnnotator^[^ ^27^ ^]^ for the functional annotation and to calculate KEGG module presence. All proteins were searched against the curated KEGG Ortholog (KO) database using Kofamscan; best matches were selected according to Kofamscan's adaptive score threshold. Proteins without KO identifiers (or matches) were extracted and searched against other databases (e.g., Swissprot, curated RefSeq database or non‐curated trEMBL database). The KO identifiers associated with all proteins in each genome (or set of proteins) were extracted, and KEGG module presence was calculated based on the total steps in a module, the proteins (KOs) required for each step, and the KOs present in each genome. Finally, the results were compiled in a single matrix‐like module presence table for all genomes.

### Statistical Analysis

The association of niche breadth of the ASVs with metabolic pathways and functional genes of representative genome by fitting generalized linear models with a binomial distribution and using the logit as a link function with core R functions was determined.^[^ ^39^ ^]^ For each binarized functional gene, a single model with the presence/absence of that gene type as the response variable and the niche breadth of each genome as the independent variable was fitted. For each metabolic pathway, module presence as a binarized variable was defined. A completeness value of “1” indicates that some genes in a pathway module were present, “0” indicates that no genes were found in this pathway module. Then a single model with the 0/1 of that metabolic module as the response variable and the niche breadth of each genome as the independent variable was fitted. It was also identified metabolic pathways that had the significant association with bacterial niche breadth of MAT, soil moisture, soil nutrients (AP, NO_3_ and NH_4_ and SOM) and pH. The statistical significance of the model coefficients using the Wald test as implemented with R core functions was evaluated. The slope (positive or negative) of the relationship between niche breadth and the presence/absence of each gene and completeness of metabolic pathway from the model estimates was obtained. Finally, those functional genes and metabolic pathways with the same significant association (*P* < 0.05 and same positive or negative direction of the model estimate) that occurred in two or three ecosystems with consistent patterns to be associated with bacterial niche breadth was retained. To define the bacterial dimensionality of trait space and identify the dominant axes of variation across all 55 metabolic modules identified in Figure 4b, it was conducted principal component analysis (PCA). Respective loadings were assessed through the correlation of individual metabolic modules with PC1. It was also further examined interrelatedness between metabolic modules by performing pairwise Spearman correlations, clustering correlation coefficients (hierarchical clustering; Ward) and estimated the number of discrete clusters of metabolic pathways using the maximal average silhouette width.^[^ ^67^, ^68^ ^]^

The association between life‐history strategies and niche breadth from two perspectives was tested: the average completeness and Pielou evenness of **m** odules aggregated within Life **H** istory **S** trategies (mLHS) (Figure 6). The average mLHS for each ASV was calculated as the mean completeness of modules involved with life‐history strategies. The positive association between average of life‐history strategies and niche breadth indicates the higher the module completeness, the broader the niche of bacterial taxa. The evenness mLHS for each ASV was calculated as the Pielou evenness of life‐history strategies calculated by the mean of module completeness. Life‐history strategies evenness describes the commonness or rarity of a life‐history strategy and refers to how close in completeness for each life‐history strategy. The higher the Pielou evenness, the closer each life history strategy was to completeness, indicating that the taxon maximize a diversity of traits associated with different life history strategies.

## Conflict of Interest

The authors declare no conflict of interest.

## Author Contributions

All authors contributed intellectual input and assistance to this study and the manuscript preparation. Z.P. conducted the experiments, analyzed the data, and wrote the manuscript. S.J. and G.W. conceived and designed the experiments, and revised the manuscript. Y.Z. and X.Q. contributed to genomic data. X.L., H.G., Y.L. and Y.A. contributed to survey collection and sample processing.

## Data Availability

The raw data that support the findings of this study are openly available in the Beijing Institute of Genomics (BIG) Data Center BioProject PRJCA020242.

## Code Availability

All data analyses were performed in R 4.1.3 (www.R‐project.org). The R script is available in a publicly accessible database (https://github.com/Pong2021/bacterial‐niche‐breadth).

## Supporting information



Supporting Information

## Data Availability

The data that support the findings of this study are available on request from the corresponding author. The data are not publicly available due to privacy or ethical restrictions.
