# Supplementary material for: Trait‐Based Life History Strategies Shape Bacterial Niche Breadth
Source: Adv Sci (Weinh). 2025 May 8;12(20):2405947. doi: 10.1002/advs.202405947 (PMC12120777; doi:10.1002/advs.202405947)
Supplement: Supplementary file 1 — Supporting Information [file ADVS-12-2405947-s001.docx]

Supplementary Materials for

**Trait-based Life History Strategies Shape Bacterial Niche Breadth**

Ziheng Peng *et al.*

**This PDF file includes:**

Figs. S1-S15

Tables. S1-S2


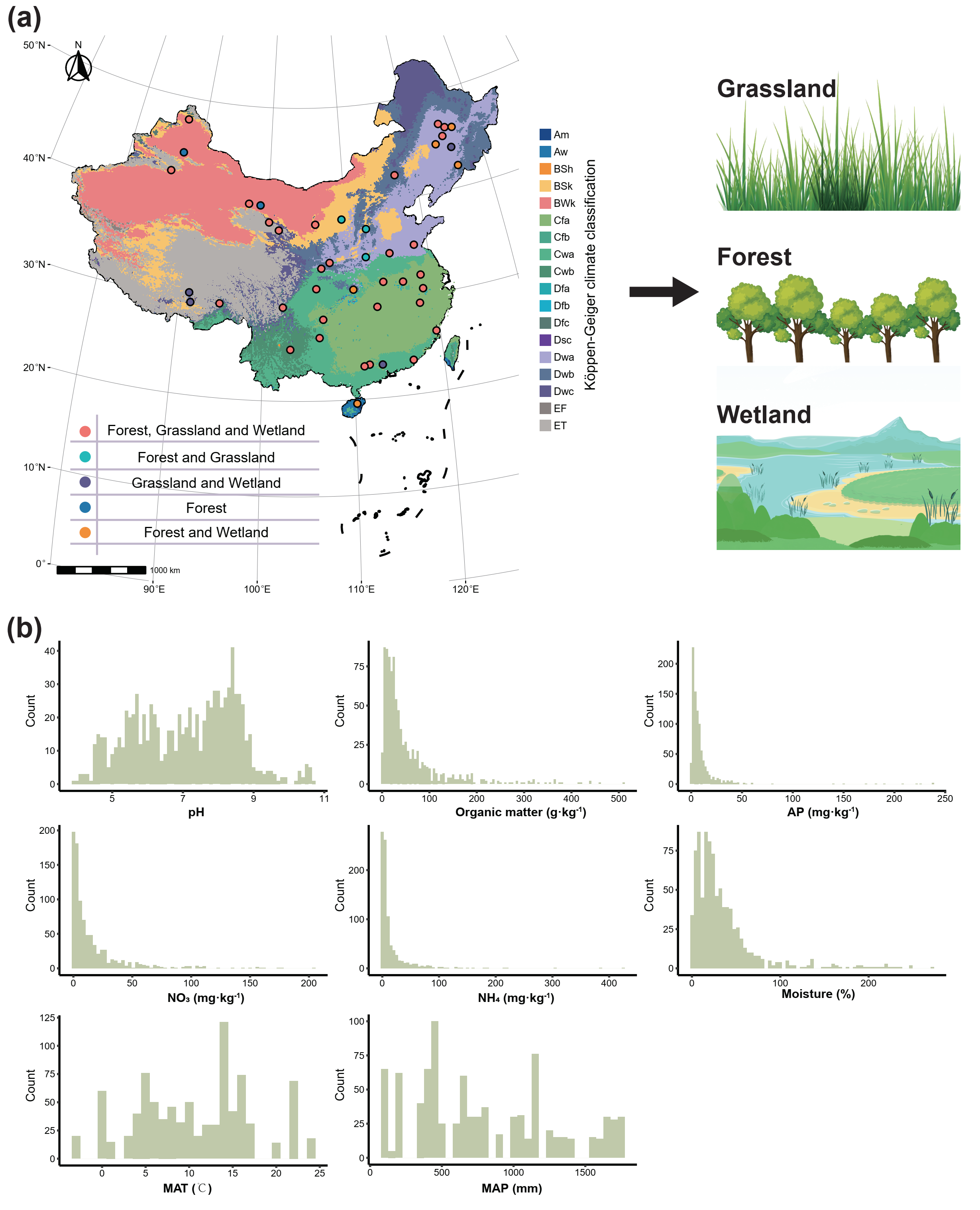
**Fig. S1 Distribution of study sites and environmental variables.** (a) Map showing 44 regions covering forests, grasslands and wetlands. (b) Distribution of a total of 893 soil samples across eight environmental variables used for this study.
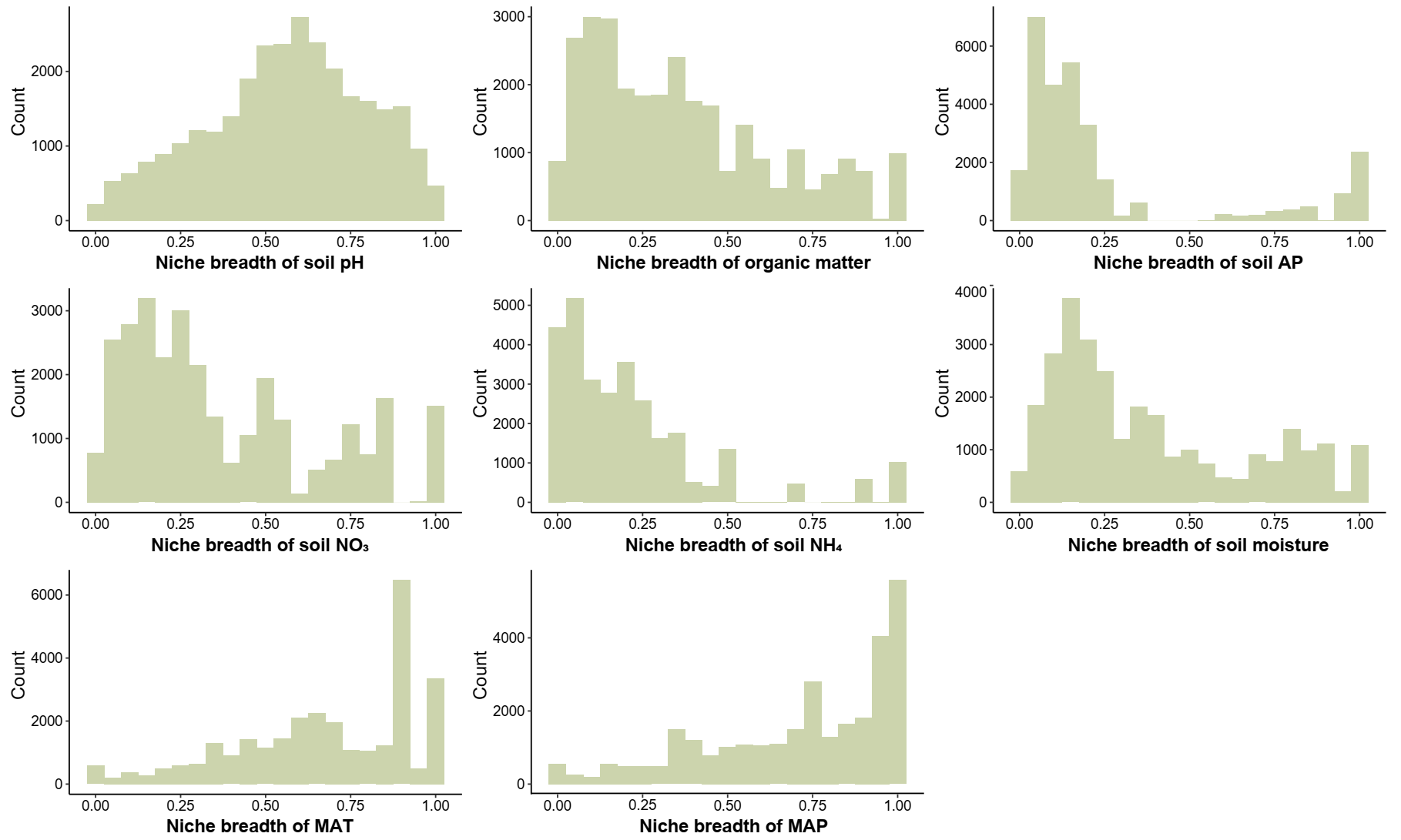


**Fig. S2 Niche breadth of bacterial taxa across eight environmental variables.** Soil AP, soil available phosphorus; Soil NO_3_, soil nitrate nitrogen; Soil NH_4_, soil ammonium nitrogen; MAT, mean annual temperature; MAP, mean annual precipitation.


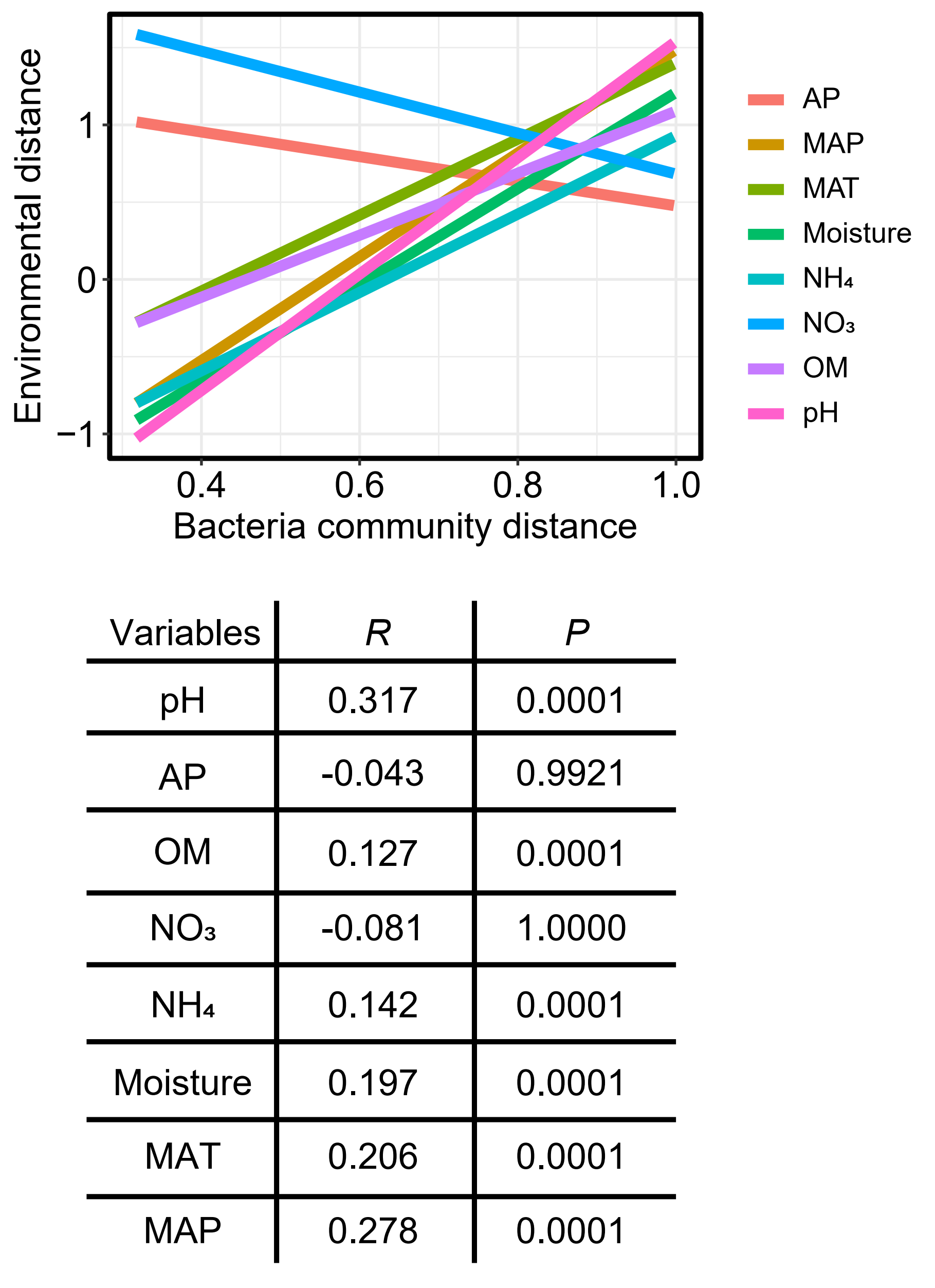


**Fig. S3 Mantel tests with 9999 permutations between eight environmental variables and bacterial community composition (pairwise Bray-Curtis distances).**


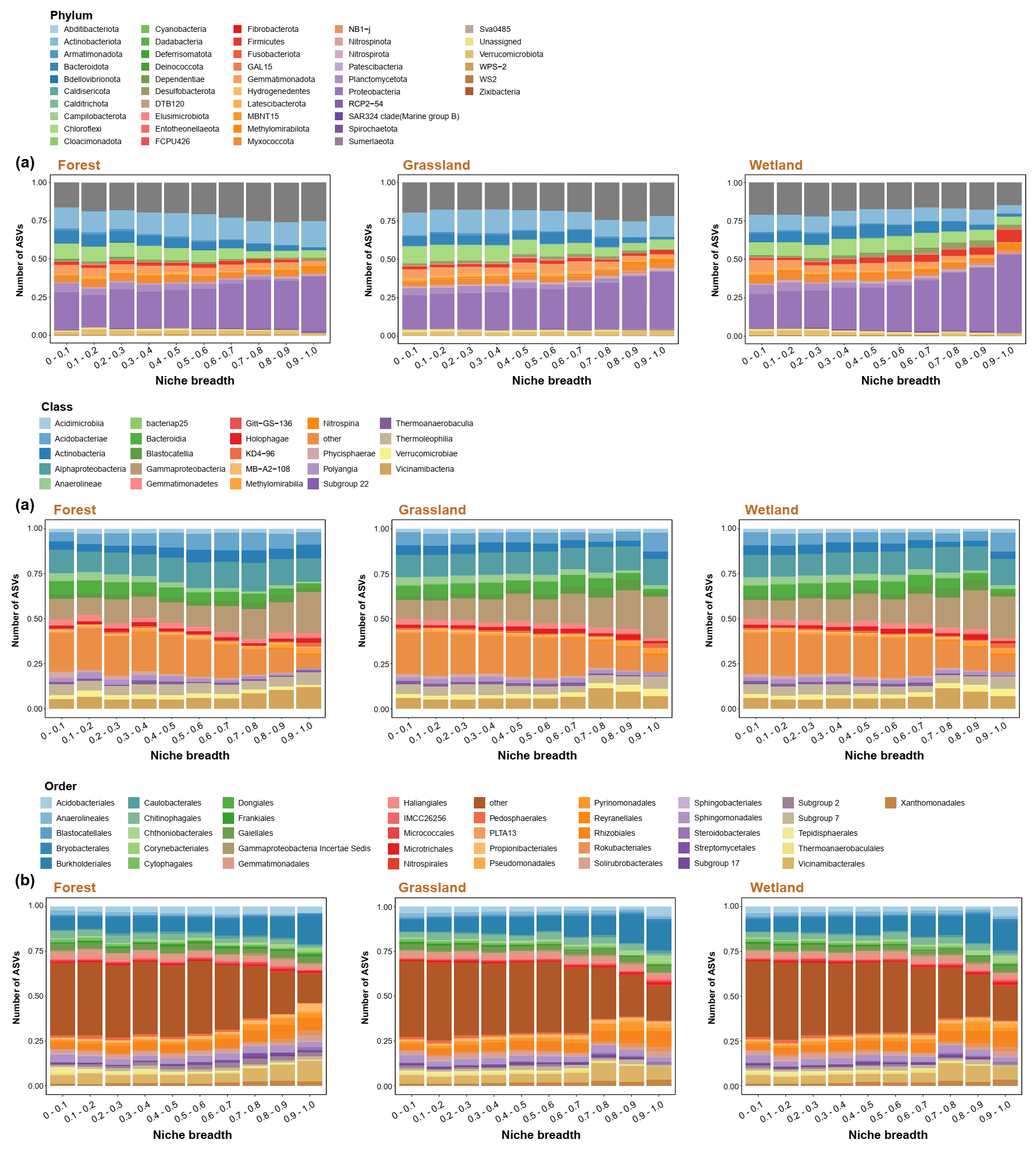


**Fig. S4 Relative abundance of the ASVs with niche breadth of soil bacteria classified at the phylum level (a), class level (b) and order level (c).**


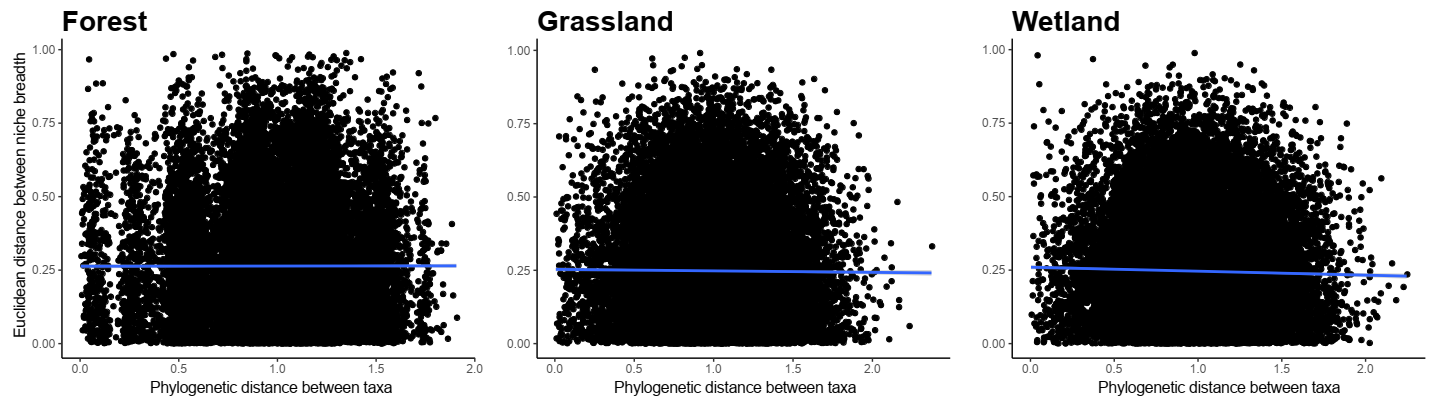


**Fig. S5 Low phylogenetic signal of niche breadth in soil bacteria.** The relationship between the Euclidean distance in niche breadth and the phylogenetic distance based on branch lengths between genomes were tested as non-significant via Pearson’s correlation.


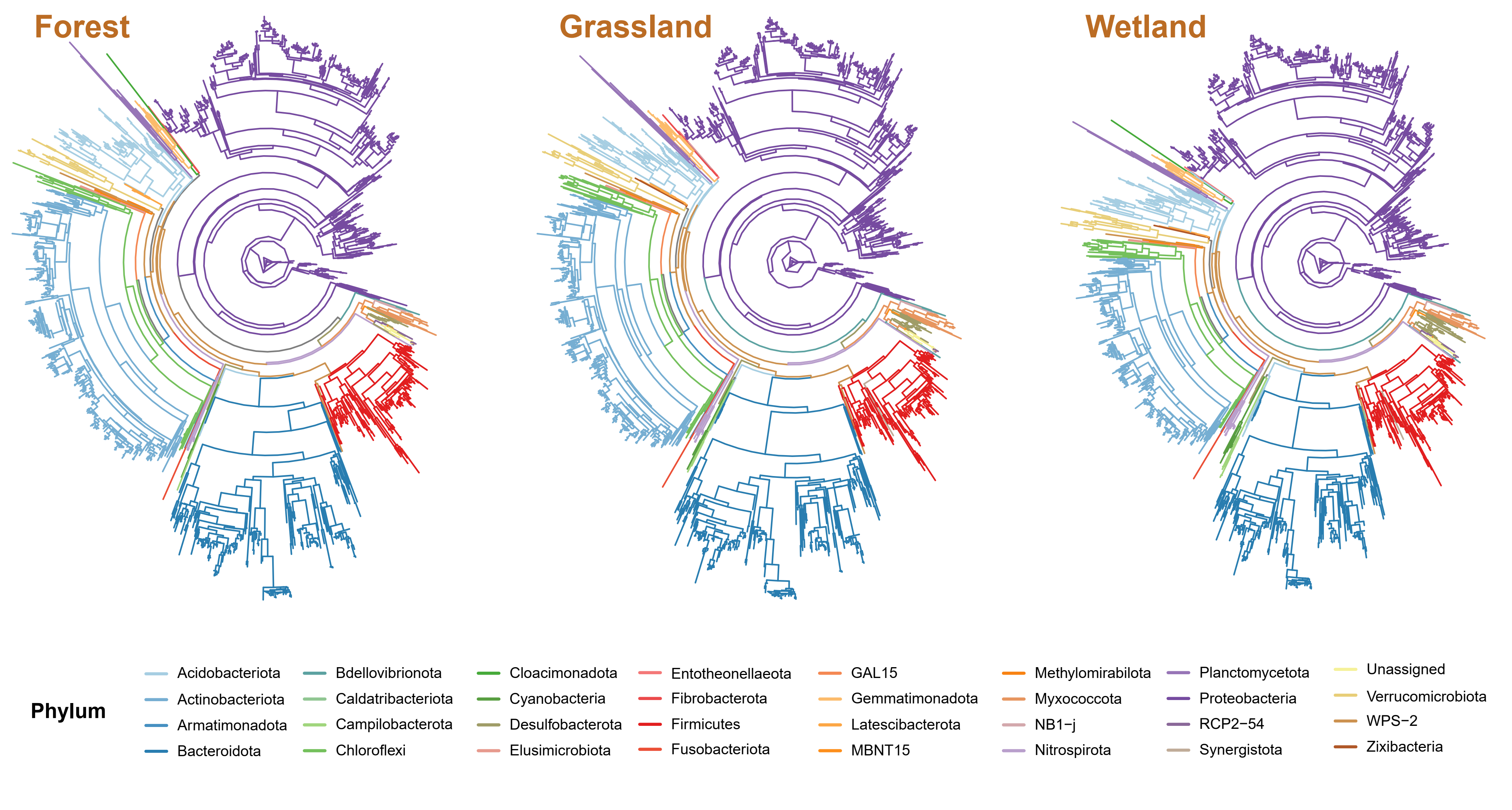


**Fig. S6 Taxonomic distribution of soil bacteria with representative genomes in the GTDB dataset.** A total of 1657, 1775 and 1894 bacterial taxa with representative genomes were shown in forests, grasslands, and wetlands, respectively. These bacterial taxa covered 31 phyla, which represents a wide of taxonomic and phylogenetic distributions.


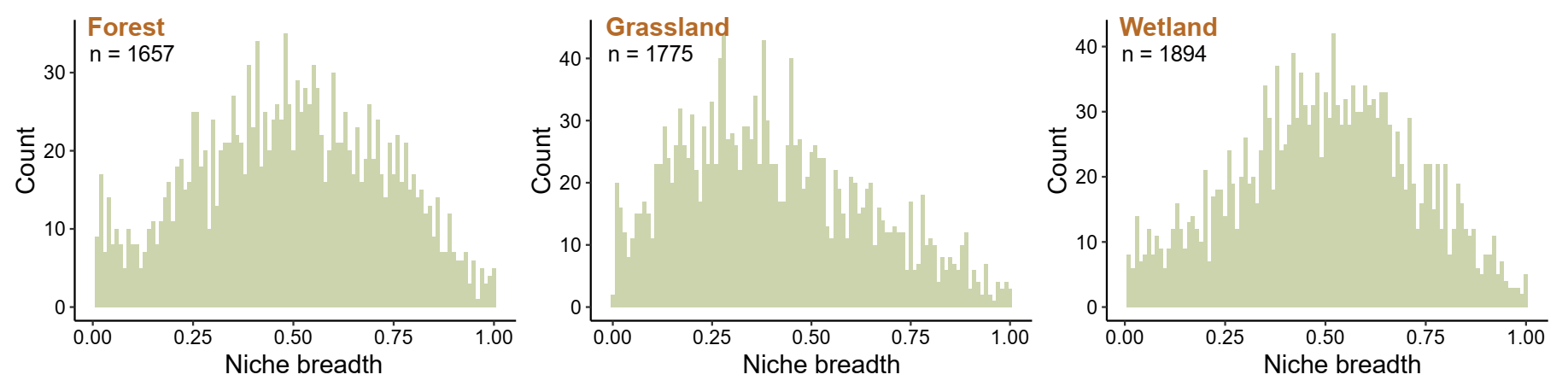


**Fig. S7 Distribution of niche breadth of bacterial taxa with representative genomes in the GTDB dataset.** A total of 1657, 1775 and 1894 bacterial taxa with representative genomes were shown in forests, grasslands, and wetlands, respectively.


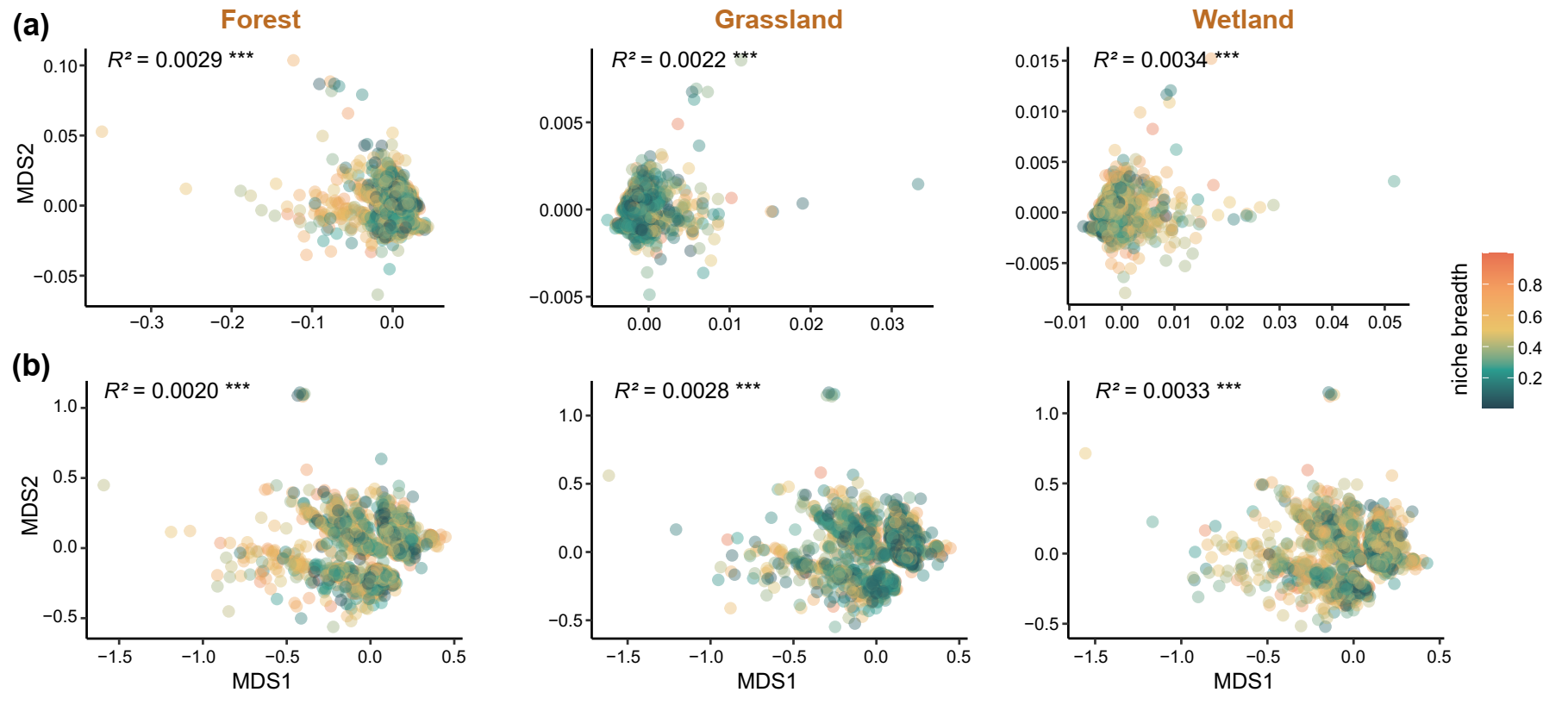


**Fig. S8 Non-metric multidimensional scaling (NMDS) ordinations based on Bray–Curtis dissimilarity showing KEGG modules (a) and KOs (b) across niche breadth of soil bacteria.** Permutational multivariate analyses of variance (PERMANOVAs) were conducted to calculate the variance associated with niche breadth, with *R^2^* values and their significance given in the top left-hand corner (***, *p* < 0.001).


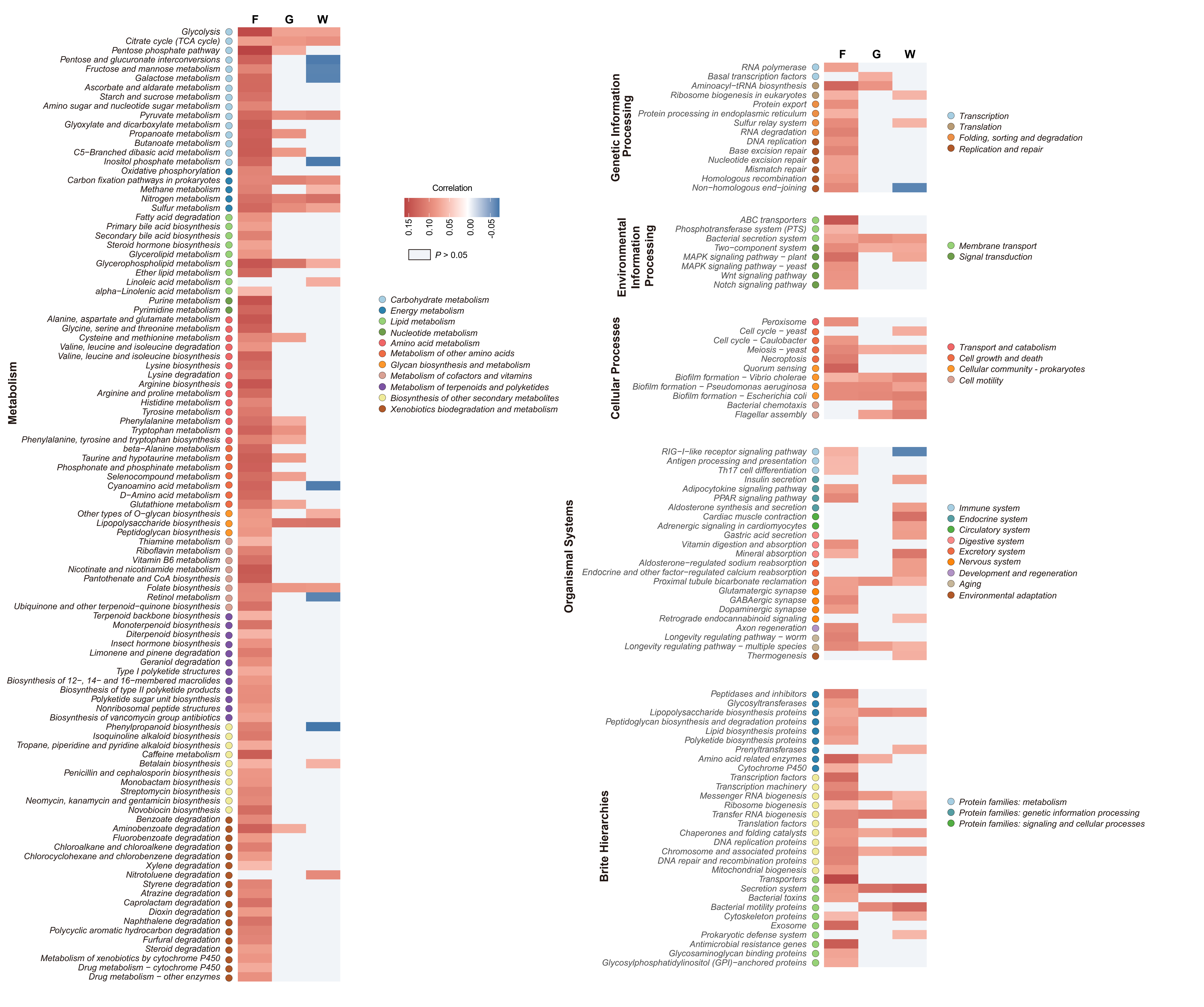


**Fig. S9 Associations between the total number of gene copy belonging to each category at Level 3 of KEGG categories and bacterial niche breadth in three ecosystems.** Those functional categories that are significantly associated with niche breadth in at least one ecosystem are shown. F, forest; G, grassland; W, wetland.


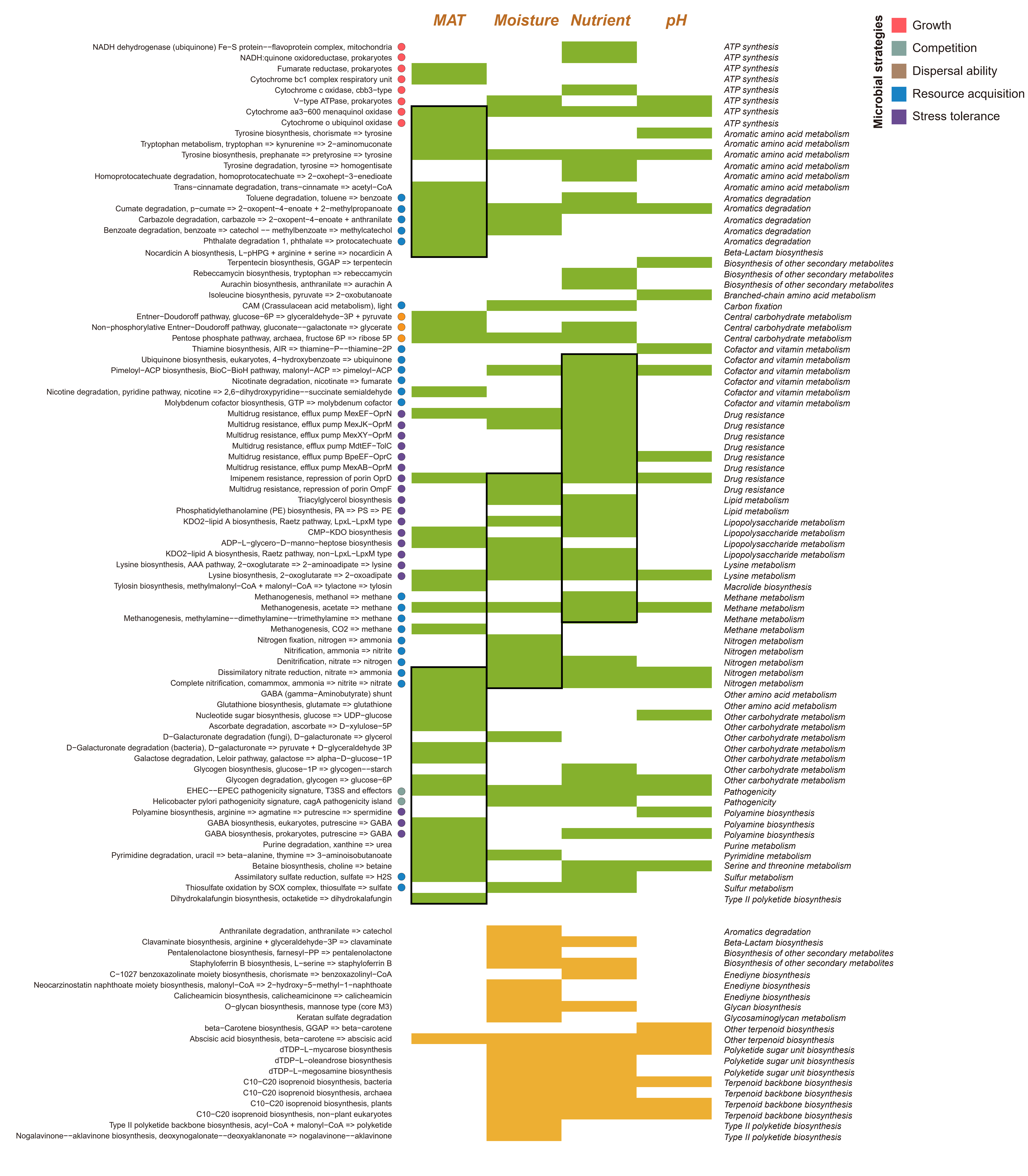


**Fig. S10 The relationships between metabolic pathways and niche breadth across MAT, soil moisture, soil nutrients and pH.** Positive trends (green) indicate that the completeness of metabolic pathways is associated with wider niche breadth, and negative trends (yellow) indicate that the completeness of metabolic pathways is associated with narrower niche breadth. We only included metabolic pathways that had shared relationships to niche breadth in at least two ecosystems. Metabolic pathways belonging to the five life history strategies are highlighted in different colors. MAT, mean annual temperature.


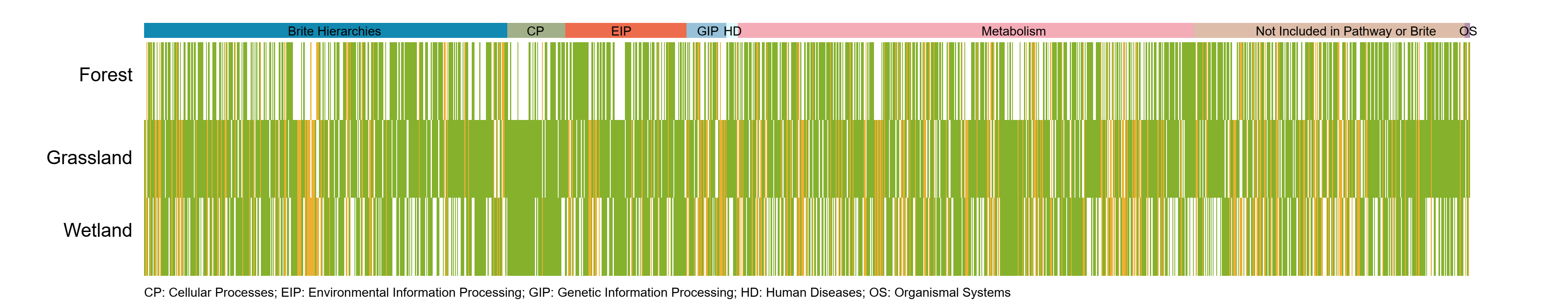


**Fig. S11 Associations between KOs and bacterial niche breadth in three ecosystems.** Green and yellow represent positive and negative relationships, respectively. A total of 1,421 KOs that had shared relationships to niche breadth in at least two ecosystems are showed. These KOs are assigned to Level 1 of KEGG categories.


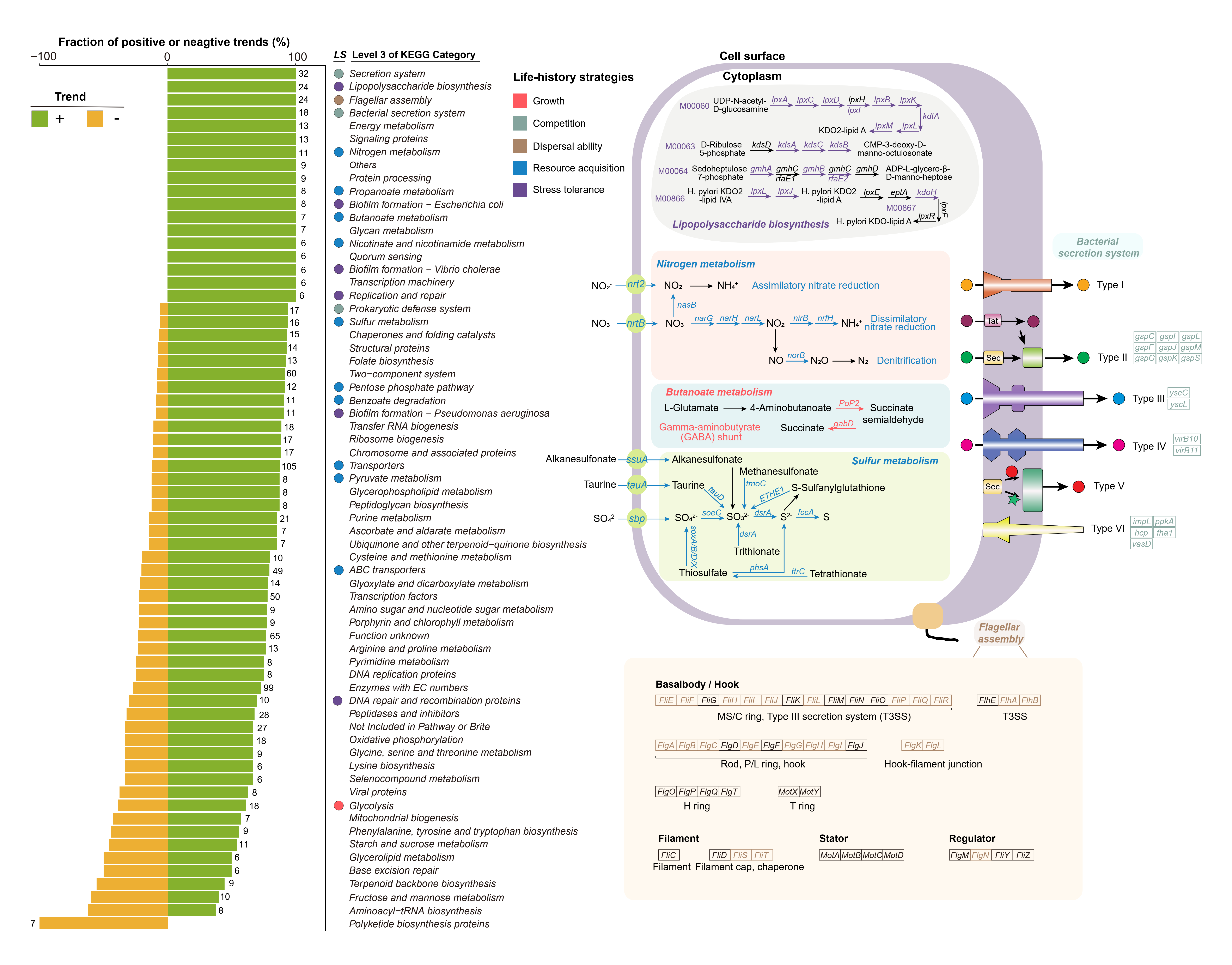


**Fig. S12 Functional genes related to bacterial niche breadth across ecosystems.** Positive trends (green) indicate that the presence of the gene is associated with wider niche breadth, and negative trends (yellow) indicate that the presence of the gene is associated with narrower niche breadth. A total of 1,421 KOs that had shared relationships to niche breadth in at least two ecosystems belonged to 202 categories in level three of KEGG functional categories, of which 66 categories containing at least six KOs are shown. The number at the right and left of the barplot correspond to the number of KOs with significant relationship with niche breadth in each level three of KEGG functional category. Functional genes belonging to the five life history strategies are highlighted in different colors. Several representative categories and genes that are positive with niche breadth are visualized, e.g., flagellar assembly, bacterial secretion system, lipopolysaccharide biosynthesis, nitrogen metabolism, butanoate metabolism and sulfur metabolism. LS, Life-history strategies.


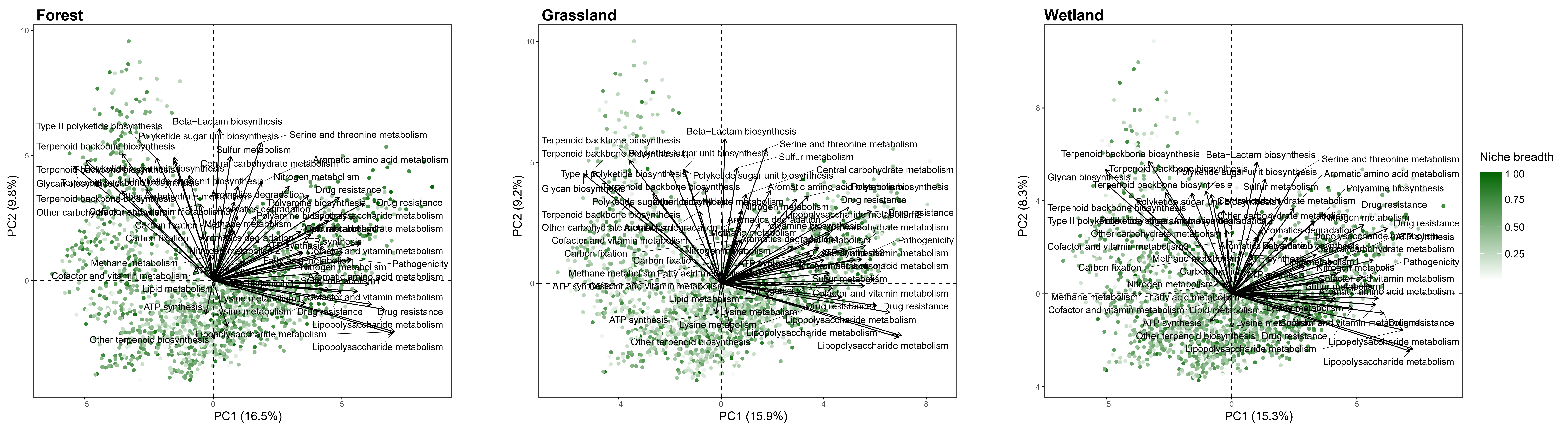


**Fig. S13 The dominant metabolic module axes and relationships.** Shown are the first two principal component axes capturing module relationships from the 55 modules (Fig. 4b).


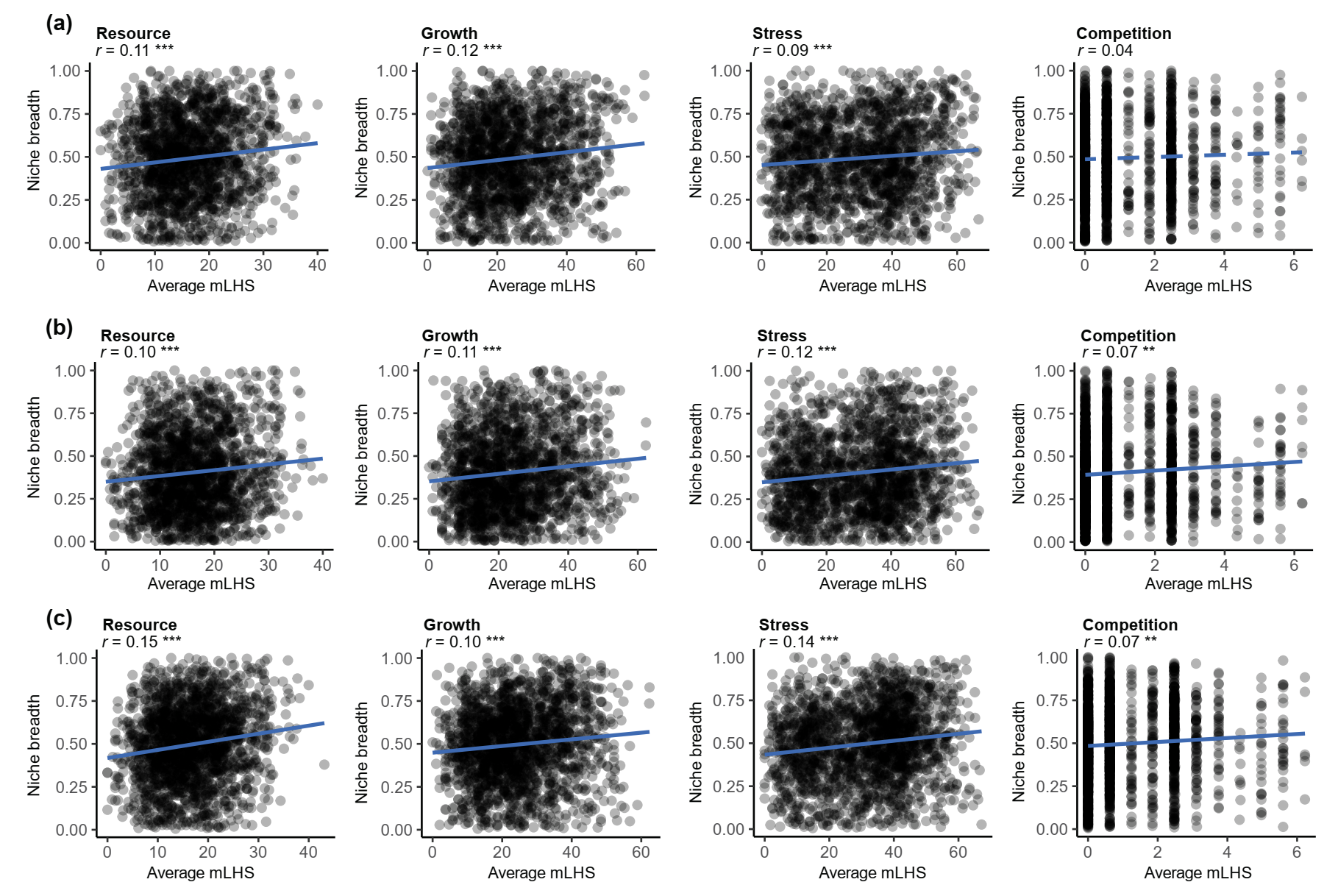


**Fig. S14 The relationships between the average completeness of modules aggregated within Life History Strategies (mLHS) and niche breadth.** The average mLHS was calculated as the mean completeness of module belonging to life-history strategies. The linear relationships were tested by Pearson correlation. Resource, Resource acquisition. Stress, Stress tolerance. *, *P* < 0.05; **, *P* < 0.01; ***, *P* < 0.001.


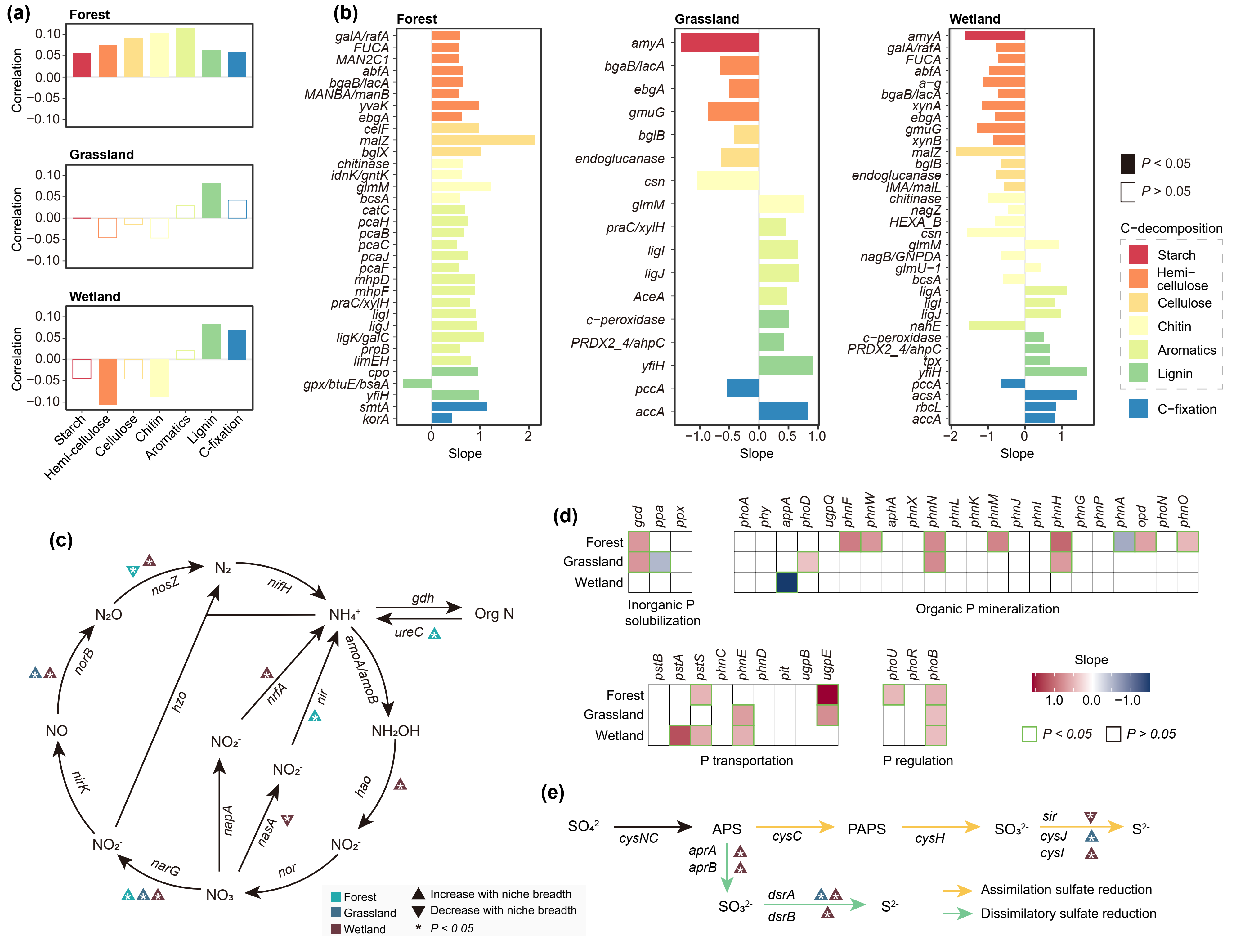


**Fig. S15 Linking bacterial niche breadth with functional genes involved in biogeochemical cycling.** (a) Bars represent Pearson’ correlation between the diversity of functional genes involved in C degradation and fixation aggregated over major substrates and bacterial niche breadth in forest, grassland and wetland soils. (b) Specific functional genes involved with C degradation and fixation with a significant slope with *P* < 0.05 are shown. (c) Genes involved in N processes where slope was significant (*P* < 0.05) are labelled in asterisks. (d) Heatmaps represent the slope calculated for each gene. Significant slope with *P* < 0.05 are highlighted with green squares. (e) Genes involved in sulfur cycling where slope was significant (*P* < 0.05) are labelled in asterisks. The slope (positive or negative) of the relationship between bacterial niche breadth and the presence/absence of each gene were obtained by fitting generalized linear models with a binomial distribution.

**Table S1. Life history traits associated with niche breadth.**

| Life-history strategies | Major traits | Related pathways |
| --- | --- | --- |
| Resource acquisition | Degradation of complex substrates | Genes for extracellular enzymes and glycoside hydrolase |
|  | Uptake of simple substrates | Transporters, siderophores |
|  | Autotrophic | Carbon fixation, methane metabolism, nitrogen fixation, photoautotraph, chemolithoautotroph |
| Stress tolerance | Biomolecular damage repair | Replication and repair |
|  | Osmolyte production | Markers for synthesis of trehalose, glycine betaine, amino acids related to osmotic stress, lysine biosynthesis, polyamine biosynthesis |
|  | Protection from desiccation | Markers for synthesis of extracellular polysaccharide, drug resistance, sporulation |
|  | Maintenance of cellular integrity | Markers for synthesis of cell walls, lipopolysaccharide metabolism |
|  | Biofilms | Biofilms |
| Dispersal ability | Chemotaxis and motility | Flagellar assembly, cell motility |
| Competition | Antibiotic production | Pathogenicity, bacterial secretion systems |
| Growth |  | Central carbon metabolism, amino acid, fatty acid, and ATP synthesis |

Notes: Traits were selected based on previous association with CSR (‘Competitor’, ‘Stress tolerant’, and ‘Ruderal’) strategies Krause et al. (18) and YAS strategies (‘Yield’,‘Resource acquisition’, and ‘Stress tolerant’) by Malik et al (12) and combination of both (17).

**Table S2. Phylogenetic signal showing the conservatism of bacterial niche breadth for eight environmental variables using Blomberg's K.** The K statistic is a measure of phylogenetic signal that compares the observed signal in a trait to the signal under a Brownian motion model of trait evolution on a phylogeny. The higher the K statistic, the more phylogenetic signal in a trait. PIC.variance.P is the quantile of the observed phylogenetically independent contrast variance versus the null distribution, which can be used as a 1-tailed P-value to test for greater phylogenetic signal than expected. Niche breadth with PIC.variance.P < 0.05 have non-random phylogenetic signal. PIC.variance.obs, Mean observed PIC variance; PIC.variance.rnd.mean, Mean random PIC variance. PIC.variance.P, P-value of observed vs. random variance of PICs; PIC.variance.Z, Z-score of observed vs. random variance of PICs.

| **Forest** | K | PIC.variance.obs | PIC.variance.rnd.mean | PIC.variance.P | PIC.variance.Z |
| --- | --- | --- | --- | --- | --- |
| pH | 2.02E-05 | 14689.32 | 27834.21 | 0.004 | -2.42837 |
| OM | 2.55E-05 | 22162.76 | 22233.4 | 0.532 | -0.0139 |
| AP | 1.62E-05 | 26090.07 | 35934.74 | 0.057 | -1.4751 |
| NO_3_ | 1.67E-05 | 31020.56 | 29966.27 | 0.563 | 0.161958 |
| NH_4_ | 3.09E-05 | 21958.59 | 22169.08 | 0.502 | -0.03218 |
| Moisture | 1.36E-05 | 29184.35 | 31605.16 | 0.352 | -0.39404 |
| MAT | 1.79E-05 | 16991.88 | 29799.78 | 0.009 | -2.108 |
| MAP | 1.63E-05 | 17266.12 | 34957.84 | 0.002 | -2.62019 |
| **Total** | **3.07E-05** | **12381.35** | **16678.02** | **0.104** | **-1.23152** |
| **Grassland** | K | PIC.variance.obs | PIC.variance.rnd.mean | PIC.variance.P | PIC.variance.Z |
| pH | 1.49E-05 | 24350.41 | 23842.14 | 0.544 | 0.098594 |
| OM | 2.26E-05 | 23896.54 | 20389.36 | 0.761 | 0.718957 |
| AP | 1.56E-05 | 41463.61 | 44819.9 | 0.387 | -0.32091 |
| NO_3_ | 2.25E-05 | 23594.88 | 31210.16 | 0.172 | -0.97464 |
| NH_4_ | 2.52E-05 | 26211.46 | 30316.13 | 0.327 | -0.47979 |
| Moisture | 6.22E-05 | 10410.04 | 12218.09 | 0.397 | -0.38922 |
| MAT | 7.59E-06 | 37241.15 | 33376.47 | 0.727 | 0.549963 |
| MAP | 9.24E-06 | 31023.76 | 31057.31 | 0.506 | -0.00494 |
| **Total** | **2.86E-05** | **15348.65** | **16590.22** | **0.394** | **-0.31764** |
| **Wetland** | K | PIC.variance.obs | PIC.variance.rnd.mean | PIC.variance.P | PIC.variance.Z |
| pH | 3.57E-05 | 10991.23 | 18556.33 | 0.053 | -1.44194 |
| OM | 3.22E-05 | 13244.6 | 23663.79 | 0.051 | -1.54814 |
| AP | 5.12E-05 | 10912.02 | 13118.19 | 0.352 | -0.47637 |
| NO_3_ | 2.11E-05 | 23521.83 | 27286.47 | 0.32 | -0.4903 |
| NH_4_ | 0.000257 | 2637.294 | 20021.4 | 0.002 | -2.38461 |
| Moisture | 2.94E-05 | 16149.78 | 26209.81 | 0.068 | -1.4432 |
| MAT | 1.26E-05 | 24714.82 | 23881.6 | 0.579 | 0.123651 |
| MAP | 9.95E-06 | 31708.01 | 25151.38 | 0.82 | 0.937341 |
| **Total** | **4.22E-05** | **9673.709** | **13133.43** | **0.181** | **-0.92108** |
